# JMJD8 Is an M2 Macrophage Biomarker, and It Associates With DNA Damage Repair to Facilitate Stemness Maintenance, Chemoresistance, and Immunosuppression in Pan-Cancer

**DOI:** 10.3389/fimmu.2022.875786

**Published:** 2022-07-11

**Authors:** Xisong Liang, Hao Zhang, Zeyu Wang, Xun Zhang, Ziyu Dai, Jian Zhang, Peng Luo, Longbo Zhang, Jason Hu, Zaoqu Liu, Changlong Bi, Quan Cheng

**Affiliations:** ^1^Department of Neurosurgery, Xiangya Hospital, Central South University, Changsha, China; ^2^National Clinical Research Center for Geriatric Disorders, Xiangya Hospital, Central South University, Changsha, China; ^3^Department of Oncology, Zhujiang Hospital, Southern Medical University, Guangzhou, China; ^4^Department of Neonatology, Yale School of Medicine, New Haven, CT, United States; ^5^Department of Interventional Radiology, The First Affiliated Hospital of Zhengzhou University, Zhengzhou, China; ^6^Department of Clinical Pharmacology, Xiangya Hospital, Central South University, Changsha, China

**Keywords:** pan-cancer, Jumonji domain containing 8, macrophage, immunosuppression, DNA damage repair (DDR), homologous recombination repair (HRR)

## Abstract

**Background:**

JMJD8 has recently been identified as a cancer-related gene, but current studies provide limited information. We aimed to clarify its roles and the potential mechanisms in pan-cancer.

**Methods:**

Pan-cancer bulk sequencing data and online web tools were applied to analyze JMJD8’s correlations with prognosis, genome instability, cancer stemness, DNA repair, and immune infiltration. Moreover, single-cell datasets, SpatialDB database, and multiple fluorescence staining were used to validate the association between JMJD8 expression and M2 macrophages. Further, we utilized ROCplotter and cMap web tool to analyze the therapeutic responses and screened JMJD8-targeted compounds, respectively, and we used AlphaFold2 and Discovery Studio to conduct JMJD8 homology modeling and molecular docking.

**Results:**

We first noticed that JMJD8 was an oncogene in many cancer types. High JMJD8 was associated with lower genome stability. We then found that high JMJD8 correlated with high expression of mismatch repair genes, stemness, homologous repair gene signature in more than 9 cancers. ESTIMATE and cytokine analyses results presented JMJD8’s association with immunosuppression. Also, immune checkpoint CD276 was positively relevant to JMJD8. Subsequently, we validated JMJD8 as the M2 macrophage marker and showed its connection with other immunosuppressive cells and CD8+ T-cell depression. Finally, potential JMJD8-targeted drugs were screened out and docked to JMJD8 protein.

**Conclusion:**

We found that JMJD8 was a novel oncogene, and it correlated with immunosuppression and DNA repair. JMJD8 was highly associated with immune checkpoint CD276 and was an M2 macrophage biomarker in many cancers. This study will reveal JMJD8’s roles in pan-cancer and its potential as a novel therapeutic target.

## Introduction

Cancers undergo various alterations during their progression, leading to a series of downstream changes, including abnormal metabolism, therapeutic resistance, unrestrained division, and weakened intercellular adhesion. These malignant phenotypes were driven by overexpressed or suppressed genes, known as oncogenes and tumor-suppressor genes, giving rise to popular cancer-gene detections. However, single cancer research restricted our global perspectives on the many faces and the potential mechanism of the target gene. Hence, pan-cancer exploration of genes has emerged as a practical approach to unraveling the mystery of cancer genes, and various integrative tools have been developed (1–5), whereas current pan-cancer studies presented limited information for lacking integrative multi-omics or polysome profile analyses.

JMJD8 belongs to the JMJD family containing a Jumonji C (JmjC) domain. This family can demethylate multiple histone or non-histone lysine sites. However, JMJD8 may not harbor the enzyme activity due to its mutations within the JmjC domain, and no enzymatic activity of JMJD8 has been found so far (6). Instead, JMJD8 has an N-terminal signal peptide, which localizes it to the endoplasmic reticulum as a luminal endoplasmic reticulum protein, making JMJD8 involved in protein folding *via* interacting with other factors (7). JMJD8 interacted with other proteins to form oligomers or complexes (7). For instance, JMJD8 interacted with PKM2 by direct binding (8) to accelerate the glycolysis rate of endothelial cells and promote the angiogenic sprouting process, indicating its non-enzyme function to regulate processes within cells.

JMJD8 was closely related to cancer activities. JMJD8 was first identified as a tumor suppressor whose knockdown promoted DNA double-strand breaks repair and enhanced the proliferation of non-small lung cancer (NSCLC) and osteosarcoma cell lines (9). However, another study has demonstrated JMJD8 as an oncogene, as it facilitated EGFR stability and promoted the proliferation and invasion of NSCLC (10). Similarly, in colorectal cancer (CRC), JMJD8 was also reported to boost cancer proliferation and invasion (11). The role of the novel identified gene JMJD8 in cancer progression remains controversial.

DNA repair enables cells to fix the broken DNA after physical or chemical damage. DNA repair relies on many fixing systems, and one of the essential systems is homologous recombination repair (HRR) (12). DNA damages signal propagates along the chromatin and triggers the chromatin remodeling (13); this activates the HRR-related pathway to repair broken double chains. When base mismatching occurs, the mismatch repair (MMR) pathways are activated to correct the errors, and cancers utilize MMR and HRR to maintain genome stability, stemness, and chemoresistance (14, 15). JMJD8 was previously reported to affect DNA repair genes, but pan-cancer evidence is required for further exploration (9).

The tumor microenvironment (TME) has a critical role in affecting tumor progression fates (16, 17); it is constructed with various immune cells and stromal cells, contributing significantly to the inhibition of the cancer progression. Nevertheless, cancers could also employ the non-cancer cells (18) to escape from immune pressure and even support their growth. Mainly, cancer-related M2 macrophages were crucial rebels (19). Therapies targeting these components to alter their immunosuppressive phenotype may improve antitumor immunity and patient prognosis (16). Though, as a novel identified biomarker, JMJD8 has been noticed to be involved in TNF-induced NF-κB pathways (20) and adipocyte-intrinsic inflammation (21), suggesting the potential interplay between JMJD8 and immune. However, currently, no clear evidence of its roles in cancer-related immunity has been presented.

**Figure 1:**
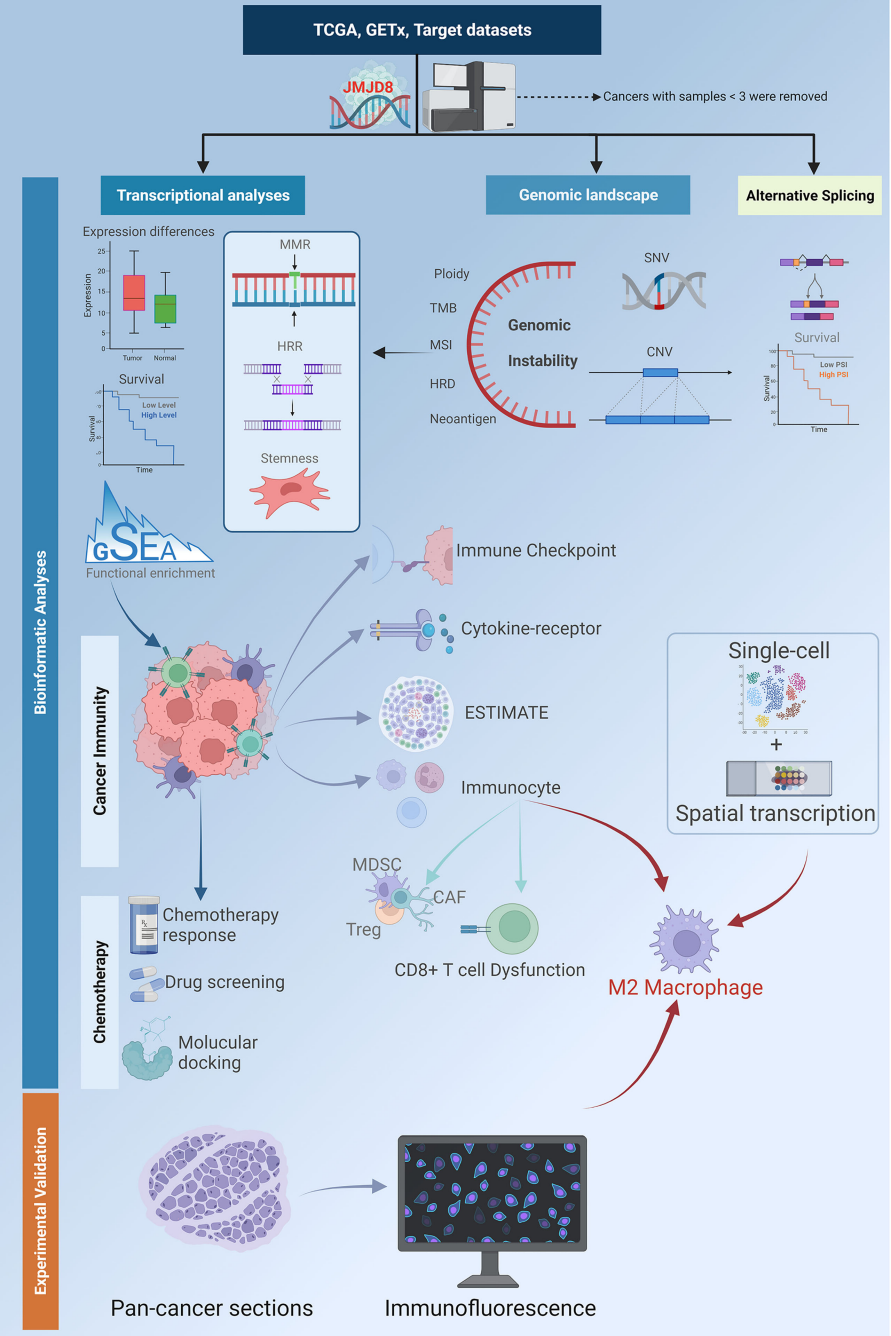
A flowchart of the study design. The flowchart describes the workflow of the study analyses. The batch-corrected, normalized expression data and clinical information of TCGA pan-cancer, GTEx, and target datasets were retrieved from UCSC. The SNV data downloaded from GDC was processed with MuTect2 software. The expression differences between cancers and non-cancer tissues, and the survival significance of JMJD8 were investigated; its association with DDR, stemness, and genome instability was discovered by transcriptional and genomic analyses. Also, the clinical value of aberrant CNV and SNV was analyzed. The functional enrichment profile identified JMJD8’s relevance with cancer immunity; this was further explored by analyzing its association with expression of the immune checkpoint, cytokines-receptors, ESTIMATE, and immunocyte infiltration. High JMJD8 was accompanied by infiltration of several immunosuppressive immunocytes, especially M2 macrophages. The association between JMJD8 and M2 macrophages was further validated by single-cell, spatial transcription data and immunofluorescence. Finally, the prediction of JMJD8-related chemotherapeutic responses and molecular docking of potential drugs were performed. This flowchart was created by BioRender.com.

In this study, we conducted a poly-omics pan-cancer exploration of JMJD8 by various integrative analyzing tools and the samples collected from cancer and normal tissue databases to reveal its correlations with clinical features, multi-omics heterogeneity, and particularly its roles in DNA repair and cancer immunity (**Figure 1**). We also conducted multiple fluorescence staining to confirm the expression of JMJD8 in M2 macrophages. Finally, the JMJD8-targeted drugs were sought for specific cancers. These presented a comprehensive understanding of JMJD8’s roles in cancers and will provide clues for developing novel targeted therapy.

## Materials and Methods

### Data Access and Procession

The batch-corrected, normalized pan-cancer and normal tissue datasets were obtained from the University of California, Santa Cruz (UCSC) datasets, including The Cancer Genome Atlas (TCGA; containing 33 cancer types), Therapeutically Applicable Research to Generate Effective Treatments (TARGET; containing 7 pediatric cancers), and the Genotype-Tissue Expression (GTEx), composed of 54 normal tissues). Cancer types with less than 3 samples were excluded. The simple-nucleotide variation (SNV) data processed with Mu Tect2 software (22) were downloaded from the GDC data portal (https://portal.gdc.cancer.gov/).

The single-cell sequencing datasets of bladder urothelial carcinoma (BLCA) (GSE145137), cholangiocarcinoma (CHOL) (GSE125449), glioblastoma multiforme (GBM) (GSE138794), head and neck squamous cell carcinoma (HNSC) (GSE103322), kidney renal clear cell carcinoma (KIRC) (GSE121636 and GSE171306), liver hepatocellular carcinoma (LIHC) (GSE125449), and ovarian serous cystadenocarcinoma (OV) (GSE118828) were collected from Gene Expression Omnibus (GEO) (https://www.ncbi.nlm.nih.gov/geo/), and lung adenocarcinoma (LUAD) single-cell dataset was downloaded from the National Center for Biotechnology Information (NCBI) BioProject #PRJNA591860. The FASTA sequences of JMJD8 protein were obtained from the NCBI protein database. The molecule structure data were downloaded from the free dataset ZINC (https://zinc15.docking.org/).

### Differential Expression of JMJD8 in Normal, Cancerous, and Different-Stage Tissues and Risk Groups

The JMJD8-associated diseases or phenotypes were first presented with a bubble graph from the Open Target Platform (https://platform.opentargets.org/). The JMJD8 mRNA expression differences between cancer tissues and adjacent normal tissues were analyzed in the Gene_DE module of TIMER2.0 (23) (http://timer.cistrome.org/). For the cancer types that lack enough normal adjacent samples, the GTEx and TCGA data were adopted and analyzed by the Box Plot module in the Expression DIY function of GEPIA2.0 (5) (http://gepia2.cancer-pku.cn/); the absolute log2FC cutoff and p-value cutoff were set as 0.585 and 0.01, respectively. Moreover, mRNA expression differences in cancer stages were also presented in the Stage Plot module. The Clinical Proteomic Tumor Analysis Consortium (UPTAC) data, obtained from UALCAN (1) (http://ualcan.path.uab.edu/), were used to compare the JMJD8 protein expression differences between cancer and normal tissues.

The package “survival” was run to analyze the pan-cancer prognostic risks of JMJD8, and the “survfit” function was used to analyze the survival differences and visualized by the Kaplan–Meier curves.

### Cancer-Associated Genomic Alteration and Antigen Correlation Analyses of JMJD8

Three genomic alteration types (mutation, amplification, and deep deletion) frequency in pan-cancer was analyzed *via* the Cancer Types Summary module of the online web tool cBioPortal (24) (https://www.cbioportal.org/). The processed pan-cancer SNV data were collected and combined with the protein domains retrieved from the R package “maftools” to show the mutation landscape of JMJD8. The Kaplan–Meier curves were obtained from the Copy_Number module of Tumor Immune Dysfunction and Exclusion (TIDE) (25), a web tool to study the correlations between cancer genomic or transcriptional alterations and immunotherapy responses, to explore the prognostic significance of JMJD8 copy number variations (CNVs). Moreover, the package “maftools” was used to calculate the tumor mutation burden (TMB), the microsatellite instability (MSI), homologous recombination deficiency (HRD), and neoantigen data obtained from previous studies (26, 27); the correlations between TMB, MSI, neoantigen, and the expression of JMJD8 were then presented.

### The Analyses of JMJD8's Correlation With DNA Mismatch Repair, Cancer Stemness, and Epigenetic Modification

The association between 5 mismatching repair genes (28), 4 DNA methyltransferase (29), and JMJD8 expression in pan-cancer was visualized. To further analyze the HRR signature in pan-cancer, 30 HRR-related genes were retrieved from an ARIEL3 clinical trial (30) and input into GEPIA2.0 to calculate their correlations with JMJD8. The “differentially methylated probes-based stemness index” (DMPsi) of each cancer type was also obtained from a previous study (31), and their intercorrelation with JMJD8 mRNA expression was investigated. The association between JMJD8 promoter methylation and cytotoxic T lymphocytes (CTLs) and patients’ survival rates were subsequently presented in the Methylation module of TIDE. Finally, a heatmap was plotted to display the correlation between JMJD8 and 44 N1‐methyladenosine (m1A), 5‐methylcytosine (m5C), and N6‐methyladenosine (m6A) modification genes (32–34).

### Clinically Relevant Alternative Splicing Analyses of JMJD8

To seek the clinically relevant alternative splicing (AS), the ClinicalAS module of the OncoSplicing server (4) (http://www.oncosplicing.com/) was searched for the AS events of JMJD8 included in both the SplAdder and the SpliceSeq projects. The PanPlot was displayed to show the percent spliced-in (PSI) of TCGA cancers and GTEx tissues. The PanDiff plots were presented to compare the PSI differences of the AS events (detected in more than 3 cancers) between cancers and adjacent or GTEx normal tissues. Finally, the Kaplan–Meier curves were plotted to explore the prognostic significance of the AS events in pan-cancer.

### The JMJD8 Interaction Network and Functional Enrichment Analyses

The current JMJD8 Protein–Protein Interaction network with known experiment validations was searched *via* the online web tool String (35) (https://www.string-db.org/). The pan-cancer pathway-level somatic alterations of several vital pathways were explored on UALCAN, and the expression correlations between pathway-related signature (36) and JMJD8 were investigated on GEPIA2.0. Also, the top 100 co-expression genes of JMJD8 in pan-cancer were obtained from the Similar Gene Detection function of GEPIA2.0. The top 100 genes were adopted for functional analysis of Gene Ontology (GO) with a false discovery rate (FDR) < 0.05 using the R package “clusterProfiler” the GO annotation was retrieved by R package “org.Hs.eg.db”. For the top 5 co-expression genes, JMJD8’s correlations with them were visualized by the heatmap plotted in TIMER2.0 and the scatter plots in GEPIA2.0. The quantification results of JMJD8 functional enrichment were further investigated by Gene Set Enrichment Analysis (GSEA) using the GSEA software (http://software.broadinstitute.org/gsea/index.jsp) (37). According to the median expression of JMJD8, all cancer samples were divided into low- and high-JMJD8 groups, and the gene sets c2.cp.kegg.v7.4.symbols.gmt and h.all.v7.4.symbols.gmt were downloaded from the Molecular Signatures Database (38) of http://www.gsea-msigdb.org/gsea/downloads.jsp for Kyoto Encyclopedia of Genes and Genomes (KEGG) and hallmark pathways GSEAs (p < 0.05).

### Investigation of Immunological Roles of JMJD8 in the Pan-Cancer Microenvironment

The roles of JMJD8 in pan-cancer microenvironment infiltration were first investigated by calculating the Estimation of STromal and Immune cells in MAlignant Tumour tissues using Expression data (ESTIMATE), stromal, and immune scores using the R package “ESTIMATE” (version 1.0.13) (39).

The immune checkpoint markers from a previous study (27) were obtained to analyze their correlations with JMJD8, JMJD8’s expression relevance with immune subtypes was analyzed, and its levels were compared between these subtypes in pan-cancer in the Subtype module of TISDB, a web portal for tumor and immune system interaction (3) (http://cis.hku.hk/TISIDB/). Additionally, the heatmaps were plotted to show the association between JMJD8 expression and chemokines, chemokine receptors, and immunostimulators in the Chemokine and Immunomodulator modules, respectively. To investigate cytokine treatment’s effects on JMJD8 expression, the Tumor Immune Syngeneic MOuse (40) (TISMO, http://tismo.cistrome.org/) web tool was used to compare gene expression levels across cell lines between pre- and post-cytokine-treated samples.

TIMER2.0 was used to run 5 different algorithms on the intercorrelation between M2 macrophage and JMJD8. Moreover, the SpatialDB online tool (41), a database for spatially resolved transcriptomes (https://www.spatialomics.org/SpatialDB/), was applied to analyze the spatial expression levels and overlapping of JMJD8 and M2 macrophage markers CD68 and CD163 in breast cancer and prostate cancer. For single-cell resolution, the JMJD8 expression among various cell subtypes in pan-cancer was compared with the aid of single-cell datasets collected from GEO and the Tumor Immune Single-cell Hub (TISCH) (42) (http://tisch.comp-genomics.org/). Dataset integration for KIRC was performed by the RunHarmony function from the R package "harmony". Subsequently, the immunocyte infiltrating correlations of JMJD8 were calculated by the CIBERSORT algorithm (43).

### Multiple Fluorescence Staining of Pan-Cancer Tissue Chip

Multiple fluorescence staining was performed on pan-cancer paraffin sections to validate the M2 macrophage biomarker potential of JMJD8. The sections in this study contained 9 cancer types. These sections were deparaffinized and blocked with 3% H_2_O_2_ and 2% bovine serum albumin (BSA) after antigen retrieval. Subsequently, they were incubated sequentially with the three primary antibodies, JMJD8 (mouse, 1:100, Santa Cruz, Dallas, TX, USA), CD68 (rabbit, 1:3,000, Servicebio, Wuhan, China), CD163 (rabbit, 1:3,000, ProteinTech, Wuhan, China). After primary antibody labeling, the sections were incubated by horseradish peroxidase (HRP)-conjugated secondary antibody (GB23301, GB23303, Servicebio, China), followed by tyramide signal amplification (TSA) (fluorescein isothiocyanate (FITC)-TSA, CY3-TSA, and CY5-TSA) (Servicebio, China). Next, 4′,6-diamidino-2-phenylindole dihydrochloride (DAPI) counterstaining of nuclei, the antifade mounting medium was applied, and the Pannoramic Scanner was used (3DHISTECH, Budapest, Hungary) to obtain multispectral images of the stained sections.

For fluorescence spectra, the excitation wavelength and emission wavelength for different fluorescence dyes are listed respectively as follows: DAPI (blue, 330–380 and 420 nm), CY3 (red, 510–560 and 590 nm), CY5 (pink, 608–648 and 672–712 nm), and FITC (green, 465–495 and 515–555 nm). Caseviewer (C.V 2.4) and Pannoramic viewer (P.V 1.15.3) image analysis software were used to quantify the cells with positive staining at single-cell levels in the multispectral images.

### Immunosuppressive Roles of JMJD8 in Pan-Cancer Environment

TIMER2.0 was used to present the JMJD8 expression intercorrelations with regulatory T cells (Tregs), cancer-associated fibroblasts (CAFs), myeloid-derived suppressor cells (MDSCs), and CD8+ T cells *via* different immune algorithms. Also, JMJD8’s roles in T-cell dysfunction and CTL-related prognosis in pan-cancer subtypes were investigated on TIDE.

### Analyses of JMJD8-Targeting Therapeutic Response, Compounds, and Molecular Docking

To investigate the effects of JMJD8 on the routine therapeutic response for glioblastomas and breast cancers, the JMJD8 expression differences between responders and non-responders and the receiver operating characteristic curves (ROC) of therapy-related survivals were searched on ROC plotter (44) (www.rocplot.org), an online tool to link gene expression and response to therapy using transcriptome-level data of four cancer types. The “query” tool of cMap (45) (https://clue.io/) was applied to screen out the anti-JMJD8 chemical compounds, the heatmap was plotted to show the top 30 compounds against the JMJD8-related differentially expressed gene signature, and their mechanisms of action (MoA) were also displayed. Moreover, JMJD8’s correlations with drug sensitivities were demonstrated on RNAactDrug (46) (http://bio-bigdata.hrbmu.edu.cn/RNAactDrug/), a comprehensive resource for querying associations between drug sensitivity and RNA molecules, and drugs with FDR < 0.05 were selected. The JMJD8 expression and the concentrations that cause 50% growth inhibition (GI50) of the top four cMap compounds among cell lines were analyzed using the COMPARE tool (https://nci60.cancer.gov/publiccompare/) in the Developmental Therapeutics Program (DTP) of the US National Cancer Institute (NCI).

For protein–compound interactions, homology modeling of JMJD8 protein was performed with the AlphaFold2 software (47). The rank_1 unrelaxed protein structure was estimated on SAVES v6.0 (https://saves.mbi.ucla.edu/) and applied to molecular docking. The docking was performed with the Discovery Studio software (version 4.5). After Auto Preparation of JMJD8 and ligand preparation of compounds, the binding sites, and all compound conformations were identified, the LibDock was selected for docking. The site and molecule conformation with the highest LibDockScore were determined for final interaction. The binding pocket 3D view and the intermolecular forces distance 2D view were displayed.

### Statistical Analyses

All the bioinformatics analyses were conducted on the R software. A log-rank test assessed the survival significance. The correlations were quantified by Pearson's or Spearman's correlation coefficients. The correlations between JMJD8 and methyltransferases were considered positive/negative if the statistical significance of the correlation was observed in any one of the 4 methyltransferases, and no opposite statistical result in other methyltransferases was presented. p-Values less than 0.05 (*p < 0.05) were considered significant.

## Results

### JMJD8 Is Differentially Expressed in Pan-Cancer and Can Predict the Survival of Patients

**Figure 2:**
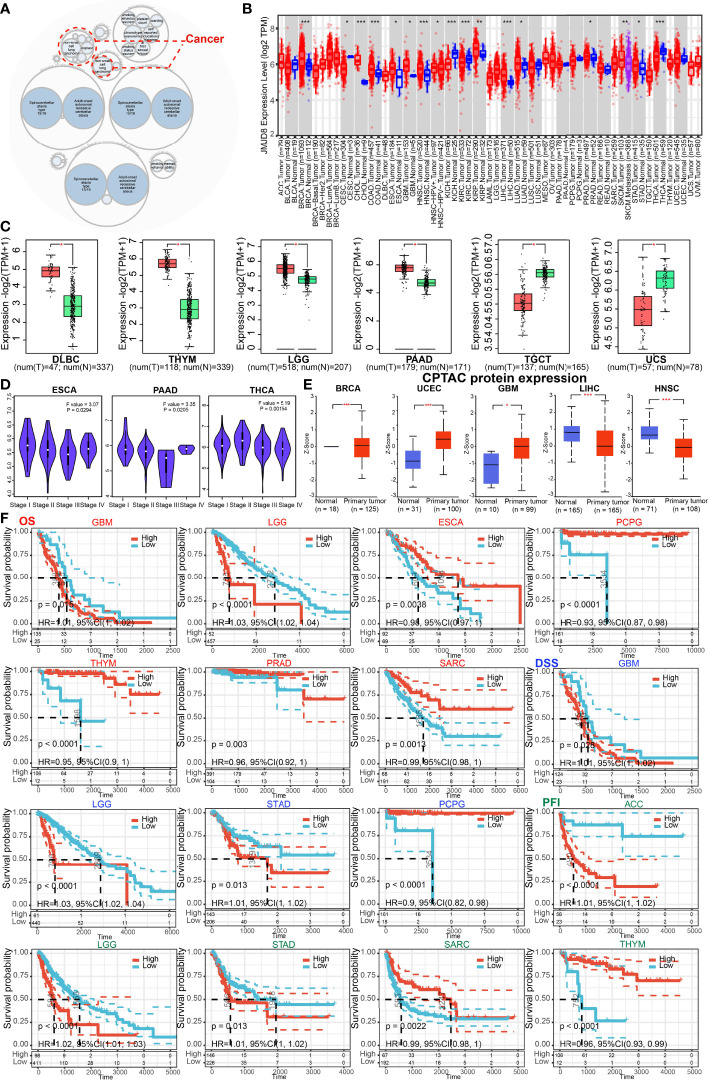
JMJD8 was differentially expressed and predicted the survival of cancers. **(A)** The diseases associated with JMJD8 were analyzed on the openTarget web tool. The red dashed lines represent JMJD8-associated cancers. **(B)** The expression levels of JMJD8 mRNA in pan-cancer, and their corresponding control tissues were analyzed on TIMER2.0. Tumors and normal tissues are colored in red and blue, respectively, and SKCM metastasis tissues are in purple. **(C)** The box plots of JMJD8 mRNA log2 expression levels between tumors and normal tissues in 6 cancer types plotted on GEPIA2.0. T and N represent tumors and normal tissues, respectively. **(D)** JMJD8 expression levels in 4 different stages of 3 cancers were also analyzed on GEPIA2.0. **(E)** The protein expression differences between normal and primary tumor tissues in 5 cancers were compared on UALCAN. **(F)** Kaplan–Meier curves are plotted to predict the OS (red), DSS (blue), and PFI (green) of TCGA patients *, **, and *** represent p < 0.05, p < 0.01, and p < 0.001, respectively.

The JMJD8-related disease was explored on OpenTarget, and the bubble graph shows that JMJD8 was associated with non-small lung carcinomas (**Figure 2A**). We then analyzed the JMJD8 mRNA level differences between pan-cancer and corresponding normal tissues on TIMER2.0; JMJD8 mRNA was significantly upregulated in 11 cancer types (BRCA, CHOL, COAD, ESCA, GBM, HNSC, KIRP, LIHC, LUAD, PRAD, and STAD) and downregulated in CESC, KICH, KIRC, and THCA (**Figure 2B**). For cancers lacking normal tissues, we compared their JMJD8 expression differences on GEPIA2.0 and UALCAN. The GEPIA2.0 results show that JMJD8 mRNA in cancers was highly expressed in DLBC, THYM, LGG, and PAAD but poorly expressed in TGCT and UCS (**Figure 2C**). Additionally, JMJD8 was negatively correlated with the high stage of ESCA, PAAD, and THCA (**Figure 2D**). The UALCAN results exhibited that JMJD8 protein was upregulated in cancers of BRCA, UCEC, and GBM and downregulated in LIHC and HNSC (**Figure 2E**). For the prognostic significance of JMJD8, forest plots of the risks (**Supplementary Material S1**) and the Kaplan–Meier curves were plotted on TCGA data. We noticed that high expression of JMJD8 was associated with lower overall survival (OS) percentages in GBM and LGG; lower disease-specific survival (DSS) percentages of GBM, LGG, and STAD; and shortening of the progression-free interval (PFI) of ACC, LGG, and STAD. By contrast, it correlated with higher OS in ESCA, PCPG, THYM, PRAD, and SARC and higher DSS in PCPG and elongation of PFI in SARC and THYM (**Figure 2F**). These results indicated that JMJD8 might be a cancer driver gene in gliomas and promote ACC progress but might be a protective gene in THYM.

### JMJD8 Gene Altered in Pan-Cancer and Correlated With Cancerous Genomic Instability and Alterations

**Figure 3:**
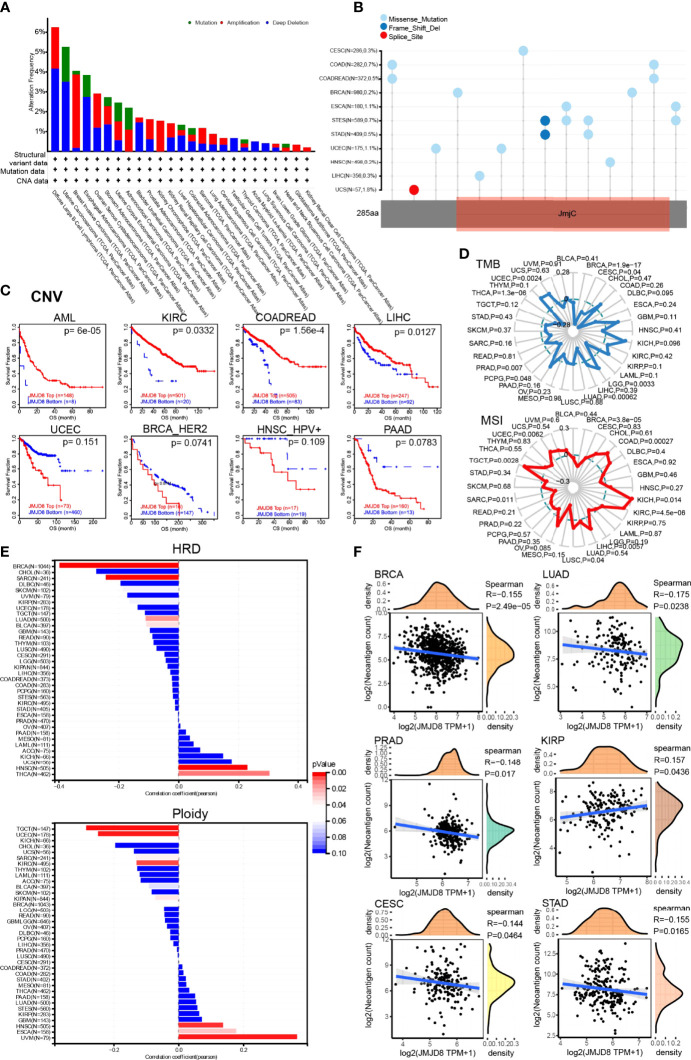
JMJD8 was associated with genomic instability in cancers. **(A)** The genomic alterations of JMJD8 in TCGA pan-cancer were analyzed, including mutation, amplification, and deep deletion. **(B)** The landscape of JMJD8 SNVs in pan-cancer, containing missense mutation, frameshift deletion, and splice site. **(C)** The Kaplan–Meier plots were drawn on TIDE web tool to show the prognostic significance of JMJD8 CNVs in 8 cancers. **(D)** The radar charts present the association between TMB (top), MSI (bottom), and JMJD8 in pan-cancer; the dashed-line circle indicates correlation coefficients of 0, intersections of solid lines (red or blue) inside the dashed-line circle represent negative correlation coefficients, and those outside the circle represent positive coefficients. **(E)** The bar chart shows the correlation coefficients between HRD or ploidy and JMJD8 expression. **(F)** Spearman’s correlations scatter plots are presented in 6 cancers to exhibit associations between JMJD8 expression and neoantigen counts. The waves in the top and right grids mean the density of JMJD8 and neoantigen levels distribution.

Cancers characterized genomic alterations. To seek whether JMJD8 gene is altered at the genome level, we displayed the JMJD8 pan-cancer CNV and SNV analysis results and found high JMJD8 amplification in BRCA and high deep deletion rates in diffuse large B-cell lymphoma and UCS (>3%), while no high SNV rates were observed (**Figures 3A, B**). When the CNV levels were applied for patient grouping on TIDE, high JMJD8 CNV group patients showed higher survival rates in AML, KIRC, COADREAD, and LIHC but lower survival rates in UCEC, BRCA (HER2), HNSC (HPV+), and PADD (**Figure 3C**). Moreover, we compared the TMB, MSI, neoantigens, and ploidy correlations with JMJD8 in pan-cancer since these genomic alterations frequently appeared in cancers and affected patient prognosis and therapeutic responses (48–50). As displayed in **Figure 3D**, JMJD8 was positively correlated with TMB in 2 cancers (LGG and UCEC) and with MSI in 7 cancers (COAD, KICH, KIRC, LIHC, LUSC, TGCT, and UCEC). By contrast, it was negatively correlated with TMB in 6 cancers (BRCA, CESC, LUAD, PCPG, PRAD, and THCA) and with MSI only in BRCA and SARC. As for HRD, JMJD8 showed a negative correlation with it in BRCA (correlation coefficient is nearly −0.4), followed by SARC, LUAD, and BLCA, while their positive correlations were observed in THCA and HNSC. For aneuploidy, JMJD8 was positively correlated with it in 3 cancers (ESCA, HNSC, and UVM) and negatively associated with 7 cancers (TGCT, UCEC, KICH, SARC, KIRC, KIPAN, and BRCA) (**Figure 3E**). Cancer TMB and MSI often caused neoantigen presentation. As presented in **Figure 3F**, 5 cancers (BRCA, LUAD, PRAD, CESC, and STAD) showed negative associations between neoantigens and JMJD8 expression, and only KIRP presented a positive correlation. The significance threshold for all analyses was set at p < 0.05. All the results above strongly suggested that JMJD8 is a potential biomarker of genome stability in BRCA and LUAD.

### JMJD8 Correlated With Cancer DNA Repair, Stemness, and Methylation

Cancer genomic stability relied mainly on repairing the DNAs *via* different mechanisms, including DNA MMR (28) and HRR, which also contributed to stemness maintenance in cancers (12, 15). Hence, we analyzed the correlations between JMJD8 and MMR-related genes (EPCAM, MLH1, MSH2, MSH6, and PMS2), HRR signature, and cancer stemness.

**Figure 4:**
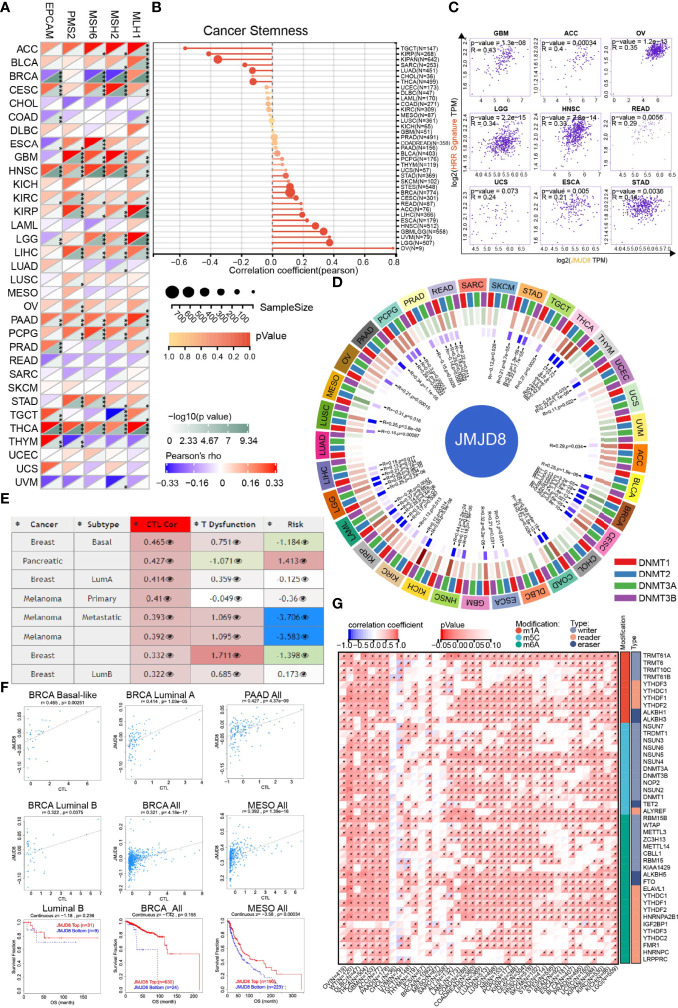
JMJD8 was involved in cancer DNA repair, stemness, and epigenetic modulations. **(A)** The heatmap displays the associations between JMJD8 and 5 MMR genes in pan-cancer. *, **, and *** represent p < 0.05, p < 0.01, and p < 0.001, respectively. **(B)** The intercorrelations between cancer stemness and JMJD8 expression are visualized in the lollipop chart, the dot size represents the sample size, and the color means the p-value. **(C)** The correlation scatter plots in 9 cancers present the correlations between the 30 genes’ HRR signature and JMJD8 expression. **(D)** The circos plot exhibits the correlations between 4 methyltransferases and JMJD8 expression. The first (outmost layer) circle refers to the pan-cancer names, and the second layer presents the four methyltransferases DNMT1, DNMT2, DNMT3A, and DNMT3B labeled by red, blue, green, and purple, respectively. The green and brown colors displayed in the third layer represent negative and positive correlation coefficient values, respectively, and the innermost blue blocks refer to the p-value (lower p-value corresponds to darker blue). **(E)** The table of correlations between JMJD8 methylation level and CTL-related factors was retrieved from the Methylation module of TIDE web tool, the third column means CTL correlation, and the fourth column refers to CTL dysfunction z-score of the interaction term. **(F)** The associations between JMJD8 methylation levels and CTL markers and the survival analyses grouped by JMJD8 high- and low-methylation are presented in the scatter and Kaplan–Meier plots. **(G)** The heatmap shows the correlation between JMJD8 expression and RNA modulations in pan-caner. * represents p < 0.05.

We discovered that JMJD8 was positively correlated with multiple MMR genes in most cancers, including ACC, CESC, GBM, HNSC, KIRC, KIRP, PAAD, LIHC, PCPG, STAD, and especially THCA (**Figure 4A**). For cancer stemness, we noticed that JMJD8 obtained strong correlations with it in OV, followed by LGG, UVM, HNSC, and ESCA. In TGCT, KIRP, and KIPAN, they exhibited negative correlations (**Figure 4B**). Unsurprisingly, 9 cancers, which showed positive correlations between stemness and JMJD8, also presented consistent trends when it came to HRR signature (**Figure 4C**), demonstrating that JMJD8 interplayed with DNA repair-mediated cancer stemness.

Since the DMPsi reflexed the DNA methylation status of cancer, we subsequently sought the JMJD8’s influences on cancer epigenetic modulations. As depicted in **Figure 4D**, JMJD8 had negative correlations with methyltransferases significantly in BRCA and other 4 cancers (MESO, PRAD, SKCM, THYM). Inversely, their positive association was exhibited in 19 cancers, including LGG, LIHC, LUSC, OV, PAAD, PCPG, STAD, TGCT, THCA, UCEC, ACC, BLCA, CESC, ESCA, GBM, HNSC, KICH, KIRC, and KIRP. Their strong positive correlations in cancers suggest that high JMJD8 promoted the promoter methylation of its target genes and suppressed their expression.

We also searched TIDE for the interplays between JMJD8 promoter methylation and cancer subtypes, CTL, and risks. **Figure 4E** presents the list of the top 8 CTL-correlated cancer subtypes; JMJD8 promoter methylation was positively correlated with CTL infiltration in BRCA, PAAD, and MESO. **Figure 4F** exhibits the correlation scatter plots and the Kaplan–Meier curves, demonstrating that JMJD8 promoter methylation was associated with CTL infiltration and predicted more prolonged survival in three BRCA subtypes and MESO.

In addition to DNA methylation, we also investigated the correlation between JMJD8 and RNA modulator gene expression. Surprisingly, we discovered that high JMJD8 was associated with a majority of RNA modulator genes in many cancers, including m1A, m5C, and m6A (**Figure 4G**), indicating that JMJD8 was involved in RNA modifications.

### Differentially Expressed JMJD8 Alternative Splicings Predicted Patient Survival

**Figure 5:**
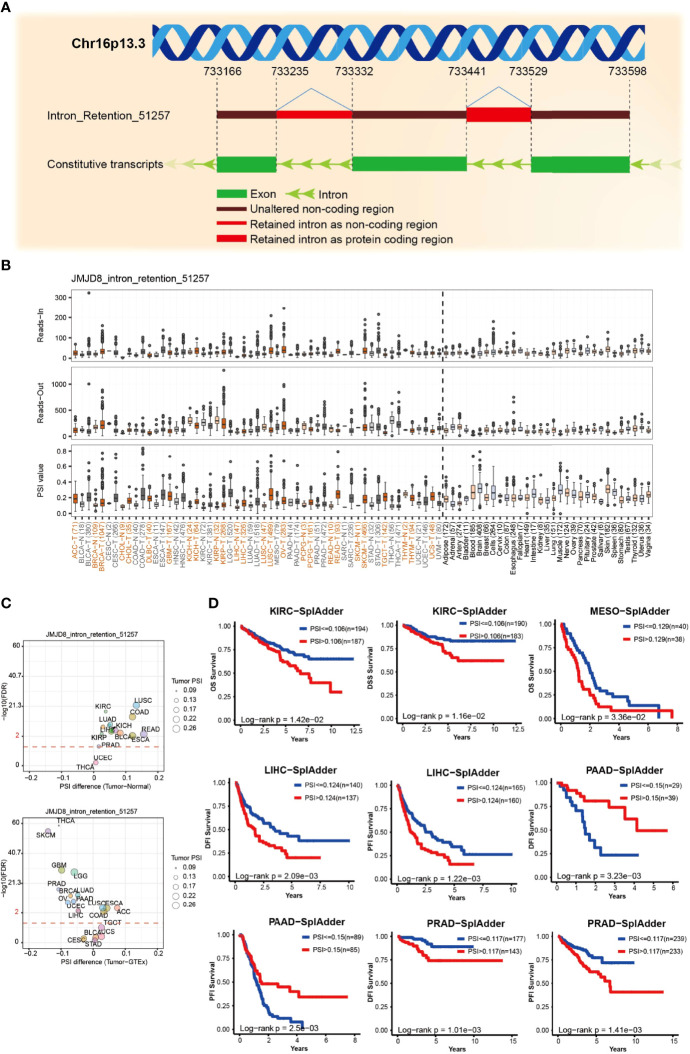
JMJD8 alternative splicing correlated to patient prognosis. **(A)** The schematic diagram of JMJD8 alternative splicing Intro_Retention_51257. **(B)** The reads-in, reads-out, and PSI value of JMJD8_ Intro_Retention_51257 in pan-cancer, adjacent, and normal tissues, respectively. The colorful labels represent cancers and their corresponding adjacent tissues, and black labels represent non-tumor tissues. **(C)** The PSI differences between tumor, adjacent normal tissues (top) and tumor, and GTEx normal tissues (bottom); red dashed line refers to 0.05 of FDR, the dot size represents the tumor PSI, and different cancers are labeled in different colors. **(D)** Kaplan–Meier curves of patients’ OS, DSS, DFI, and PFI prediction are plotted. All the data were obtained from OncoSplicing online web tool.

AS is a common post-transcriptional modification type, producing various transcripts and subsequent proteins or non-coding RNAs. Its dysregulation frequently occurs in cancers and affects tumorigenesis (51). We analyzed the ASs on OncoSplicing, 5 clinical-relevant AS events were identified, we mainly displayed the Intron_Retention_51257 event here, and the other 4 events are presented in **Supplementary Material S2**. **Figures 5A**, **B** exhibit the splicing mode and the PSI of Intron_Retention_51257 in pan-cancer; cancers such as LUSC and READ showed higher PSI than the normal samples. **Figure 5C** summarizes the statistical results of the PSI differences between tumors and normal/adjacent tissues, and those with prognostic values were presented in **Figure 5D** by the Kaplan–Meier curves. High PSI predicted lower OS and DSS in KIRC and lower OS in MESO. Similarly, high PSI also predicted lower disease-free interval (DFI) and PFI in both LIHC and PRAD. However, in PAAD, high PFI was associated with longer DFI and PFI. These results implied the biological importance of regulated JMJD8 As events in cancer progression.

### JMJD8 Was Involved in DNA Repair, Ciliary Activity, Metabolism, and Immune Pathways

**Figure 6:**
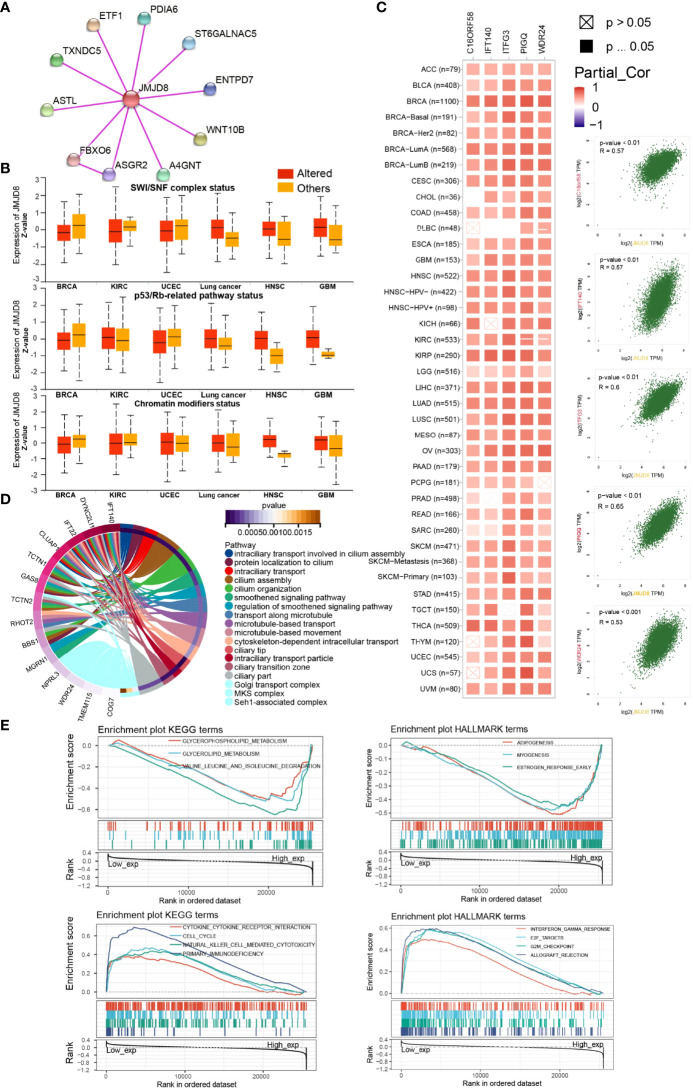
JMJD8 was involved in chromatin remodeling, cancer immunity, metabolism, and ciliary-related pathways. **(A)** Protein–Protein Interaction network of JMJD8 binding partners validated with experimental evidence. **(B)** The box plots of JMJD8 expression between pathway-level somatically altered or non-altered groups in 6 cancers were obtained from UALCAN web tool. **(C)** The correlations between JMJD8 and the top 5 JMJD8 co-expressed genes identified on GEPIA2.0 in each cancer type (left) and in all cancer samples (right). Partial_Cor means partial correlation. **(D)** The circle plots of GO pathways enriched by the top 100 JMJD8 co-expressed genes identified on GEPIA2.0. Only the top gene of each pathway is listed at the left end of the corresponding color band. **(E)** The enrichment plots of KEGG and HALLMARK terms were analyzed by GSEA in pan-cancer. The groups were divided by the median expression of JMJD8.

To investigate the functional roles of JMJD8 in cancers and the interactive or co-expressed genes, functional enrichment analyses were sequentially conducted. The interactive proteins with experimental validations were obtained from String web tool, and 10 proteins were displayed (**Figure 6A**). We then compared the JMJD8 expression between altered and non-altered pathways on UALCAN and noticed that JMJD8 was elevated in altered SWI/SNF complex, p53/Rb-related pathway, and chromatin modifiers status in HNSC and GBM while poorly expressed in BRCA (**Figure 6B**). Since somatic mutations or CNVs cannot directly explain the expression level, we explored the expression correlation between JMJD8 and these pathway-related signatures (36). We found that these signatures positively correlated to JMJD8 (**Supplementary Material S3**). The top 100 JMJD8 coexpressed genes in pan-cancer were analyzed on GEPIA2.0, and the top 5 genes (C16ORF58, IFT140, ITFG3, PIGQ, and WDR24) showed high correlations with JMJD8 in the majority of cancer types (**Figure 6C**). The functional enrichment of GO terms exhibited multiply cellular skeleton and ciliary transportation system-related activities (**Figure 6D**). Additionally, the GSEA results of GO and KEGG suggested the close association between metabolism, immune activities, and JMJD8 (**Figure 6E**).

### JMJD8 Is Involved in Cancer Immune Infiltration and Cytokine-Mediated Immune Modulations

**Figure 7:**
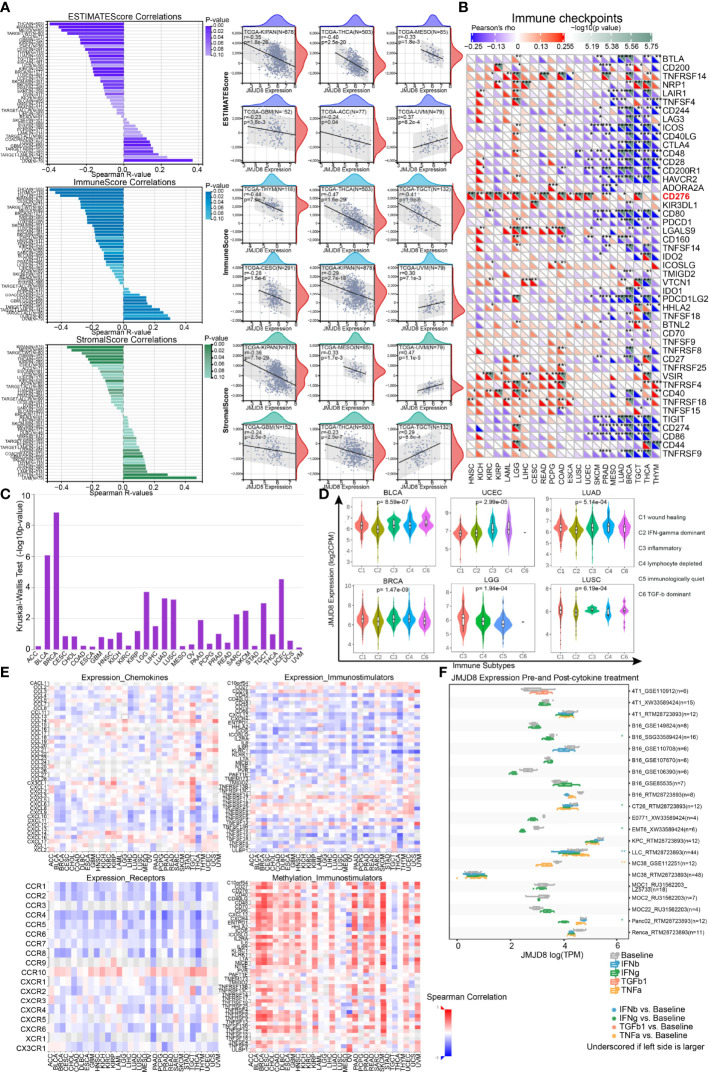
JMJD8 was reversely correlated to immune infiltration and cytokine interactions. **(A)** The bar charts of the correlations between JMJD8 and ESTIMATEScore, ImmuneScore, and StromalScore (left) are displayed, and the scatter plots of cancers with top 6 correlations are exhibited for each Score (right). **(B)** The heatmap of associations between immune checkpoints and JMJD8 expression in pan-cancer. **(C)** The correlations between JMJD8 and immune subtypes were obtained from TSIDB online tool. **(D)** The JMJD8 expression in 6 immune subtypes in 6 cancers. **(E)** The heatmaps of the correlations between JMJD8 expression and chemokines (top left), receptors (bottom left), and immunostimulators (top right) and those between JMJD8 promotor methylation levels and immunostimulators (bottom right) are presented. **(F)** The multiple box plots of cancer cell lines JMJD8 expression pre- and post-cytokine treatment were retrieved from the TISMO web tool. ESTIMATEScore, Estimation of STromal and Immune cells in MAlignant Tumor tissues using Expression data Score. *, **, *** represent p < 0.05, p < 0.01, and p < 0.001 respectively.

To investigate the immunological roles of JMJD8 in the cancer environment, we calculated the ESTIMATE of JMJD8 in pan-cancer. As depicted in **Figure 7A**, JMJD8 was reversely correlated to ESTIMATEScore and ImmuneScore in many cancers, including TCGA tumors THCA, KIPAN, MESO, ACC, GBM, BRCA, CESC, THYM, and TARGET-WT of kidney tumors. However, JMJD8 was also positively associated with them in several cancers like UVM. It presented positive relevance with JMJD8 in UVM and TGCT et al for stromal cell infiltration and negative relevance in KIPAN, MESO, THCA, KIRP, and CHOL et al. The cancers showing negative JMJD8–ImmuneScore correlations also harbored negative correlations with most immune checkpoint genes, including THYM, THCA, TGCT, BRCA, LUAD, and MESO (**Figure 7B**). However, we noticed that several markers were positively correlated with JMJD8 in many cancers. CD276 showed the highest positive correlations with JMJD8 in 15 cancers, followed by LGALS9, VSIR, and TNFRSF4. We also noticed that in LGG, JMJD8 was positively associated with as many as 24 immune checkpoint genes, indicating the involvement of JMJD8-related immune checkpoint effects.

Subsequently, we explored whether JMJD8 was differentially expressed in diverse cancer immune subtypes *via* TISIDB. The histogram exhibits that JMJD8 was significantly associated with immune subtypes in 10 cancers (**Figure 7C**), the top 6 of which are presented in **Figure 7D**; JMJD8 expression increased in the C4 subtype in BLCA, UCEC, and LUAD, implying its reverse association with lymphocyte functions. Moreover, we analyzed the associations between JMJD8 and chemokines, receptors, and immunostimulators. As visualized in heatmaps (**Figure 7E**), JMJD8 was negatively associated with several chemokines (CXCL9, 10, 11, 12, and 13), many receptors, and immunostimulators in pan-cancer. We also noticed that high JMJD8 promoter methylation was positively correlated with most immunostimulators, demonstrating that JMJD8 expression affected chemokine-mediated immunostimulations against cancers.

Finally, we compared the JMJD8 expression differences between pre- and post-cytokine treatment in cancer cell lines on web tool TISMO (**Figure 7F**). We discovered that the JMJD8 expression decreased after the IFN-γ treatment in four cell lines, and it also decreased in one IFN-β and one TNF-α posttreatment cell line.

The results from multiple perspectives demonstrated that JMJD8 is a critical factor in immunosuppressive environment construction in many cancers, probably *via* suppressing immunostimulator function and immune checkpoint effects.

### JMJD8 Is a Potential Marker of M2 Macrophage Infiltration

**Figure 8:**
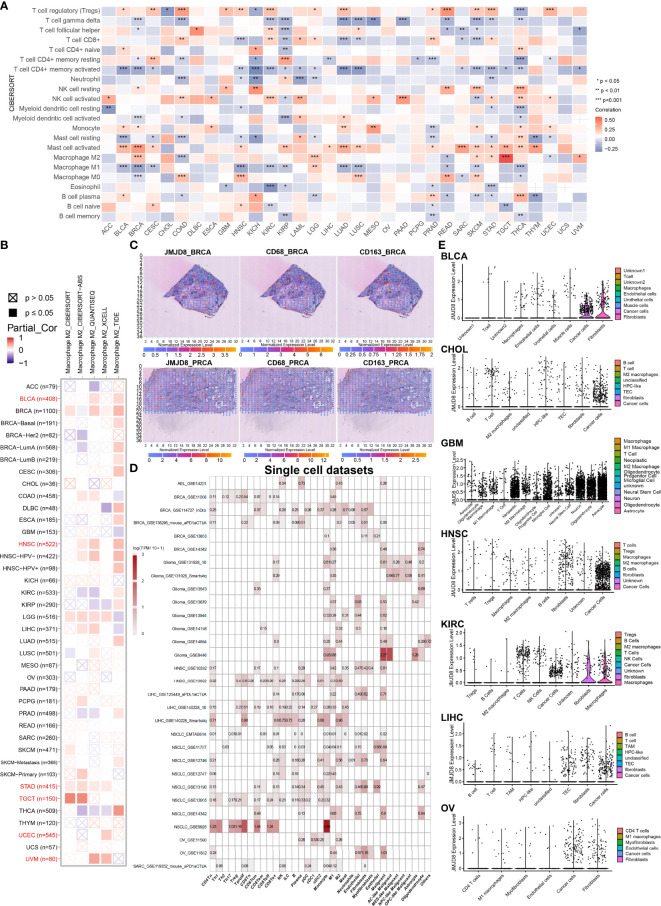
JMJD8 is a biomarker of M2 macrophage infiltration in pan-cancer. **(A)** CIBERSORT calculation of immunocyte infiltration in pan-cancer. **(B)** Multiple algorithm calculations of M2 macrophage infiltration on TIMER2.0. Partiall_Cor means partial correlation. **(C)** Spatial transcription sections show the spatial expression of JMJD8, CD68, and CD163 marker. The dot color represents the expression level of the markers. **(D)** JMJD8 expression in cancer single-cell clusters obtained from TISCH online tool. **(E)** The violin plots of JMJD8 expression in GEO pan-cancer single-cell clusters. *, **, *** represents p < 0.05, p < 0.01, and p < 0.001, respectively.

To further investigate the JMJD8 in cancer immune, we analyzed its expression in levels of immunocytes. We first ran the CIBERSORT algorithm to obtain 22 immunocyte correlations with JMJD8. We discovered that JMJD8 presented strong positive correlations with M2 macrophages in TGCT, BRCA, and LGG and negative correlations with M1 macrophages and activated CD4+ memory cells in many cancers. Also, Tregs were positively associated with JMJD8 in 13 cancers (**Figure 8A**). Focusing on M2 macrophages, we conducted multiple algorithms on TIMER2.0 to analyze the correlation between their infiltration level and JMJD8 expression in pan-cancer, and the association was observed in BLCA, HNSC, STAD, TGCT, UCEC, and UVM consistently presented. (**Figure 8B**). Spatial transcriptional data on SpatialDB were obtained to depict the spatial overlapping of JMJD8 and M2 macrophage biomarkers CD68 and CD163 on BRCA and PRCA cancer tissues (**Figure 8C**), and as expected, JMJD8, CD68, and CD163 markers presented similar spatial distributions, which implied potential co-expression of JMJD8, CD68, and CD163. What is more, we retrieved JMJD8 expression data in single-cell cellular subtypes from TISCH. As exhibited in **Figure 8D**, JMJD8 was expressed by M2 macrophages or malignant cells in AEL, BRCA, glioma, HNSC, LIHC, NSCLC, and OV. We also compared JMJD8 expression in the collected cancer single-cell datasets and discovered that M2 or undefined macrophages expressed it in BLCA, CHOL, GBM, HNSC, and LIHC (**Figure 8E**).

**Figure 9:**
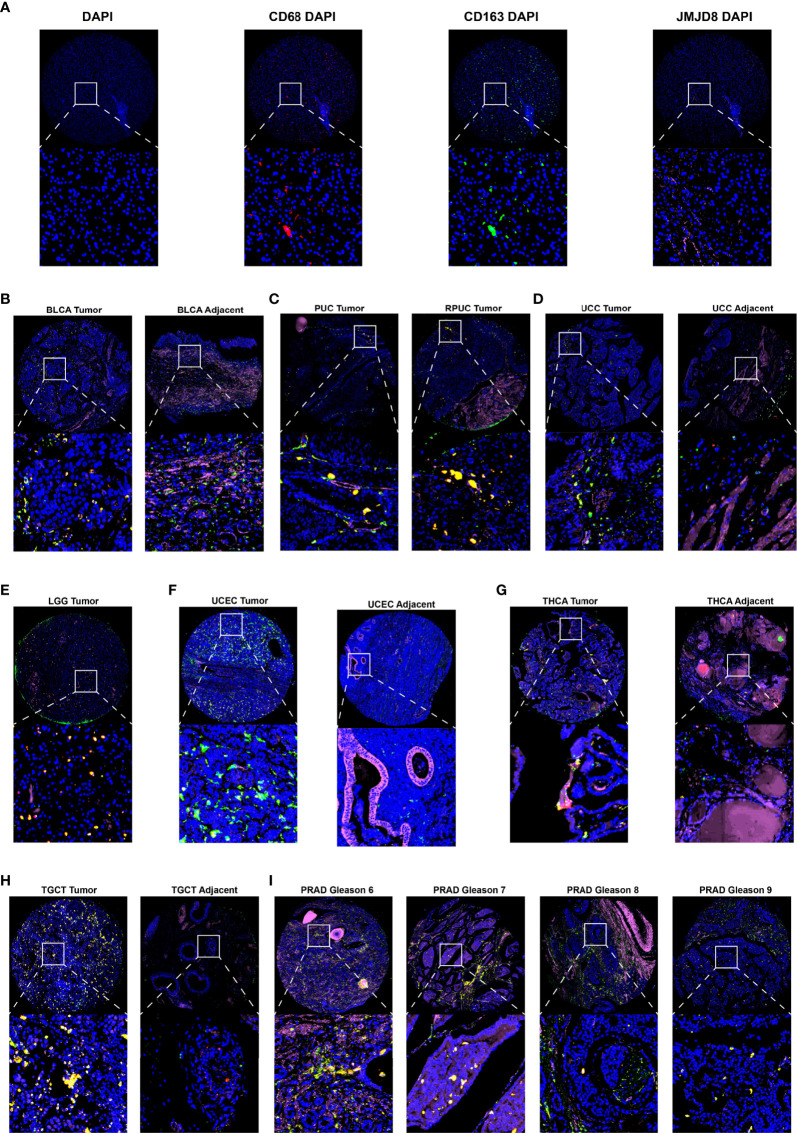
Multiple fluorescence staining of JMJD8 in pan-cancer tissue chips. **(A)** The photos show the low-magnification (top) and high-magnification views (bottom) of the single marker staining by CD68 (red), CD163 (green), and JMJD8 (pink) with DAPI and individual DAPI staining (blue). **(B–I)** The top layers show the ×10 view of the merged images of four single staining, and the bottom layers represent the local magnification of the areas within the white box in the top layer. DAPI stained nuclear in blue; CD68, CD163, and JMJD8 stained green, red, and pink, respectively.

For experimental validation, we performed multiple fluorescence (H&E staining images are presented in **Supplementary Material S4**) staining in pan-cancer paraffin sections, and the photos of JMJD8, CD68, and CD163 staining showed their co-expression in 7 cancers (BLCA, PUC/PRUC, ureter urothelial cell carcinoma (UCC), UCEC, LGG, TGCT, and PRAD) (**Figures 9A–I**). Moreover, the JMJD8 fluorescence intensity seemed higher in cancer tissues in THCA and TGCT adjacent tissues (**Figures 9G, H**), consistent with the differential expression results from TIMER2 and GEPIA2 **Figures 2B, C**. Several cancers showed positive correlations between JMJD8 and CD163 intensity, such as LGG.

From the bulk, spatial, single-cell transcriptional data and the fluorescence staining results above, we confirm the close association between JMJD8 and M2 macrophages, and these suggested that JMJD8 is a potential cancer-specific marker.

### JMJD8 Is Associated With Other Immunosuppressive Cells and Correlated With Cytotoxic T Lymphocyte Dysfunction

**Figure 10:**
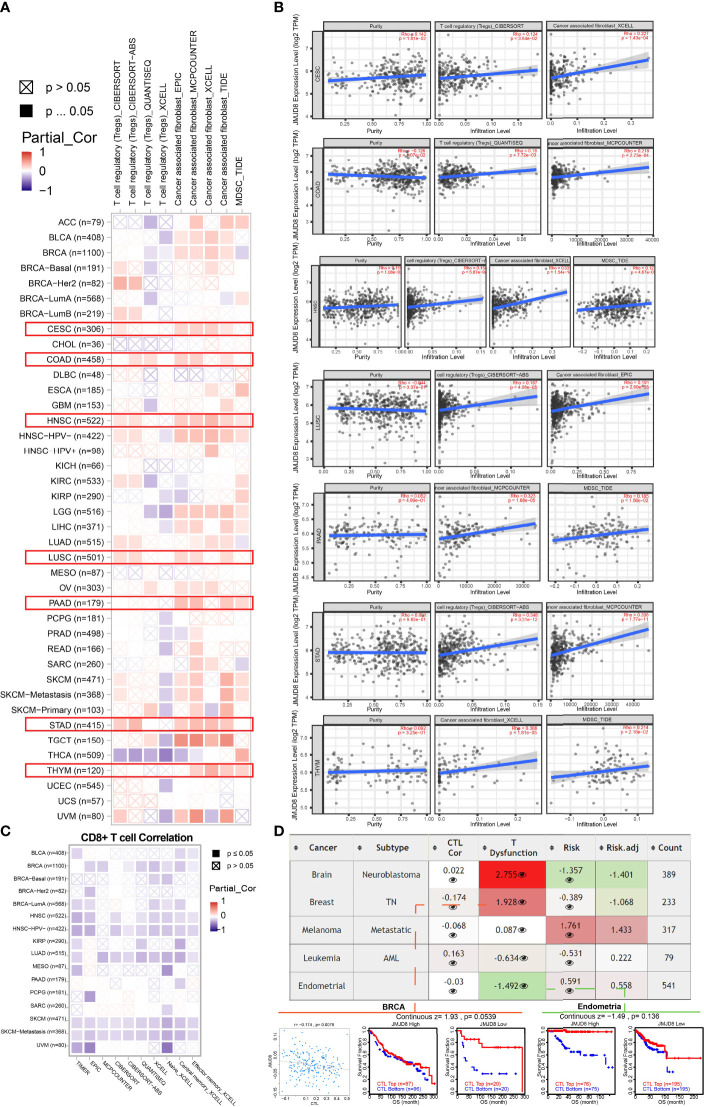
JMJD8 was correlated to Tregs, CAFs, MDSC infiltration, and CD8+ T-cell depression. **(A)** The heatmap of associations between JMJD8 level and Tregs, CAFs, and MDSC infiltration was calculated by multiple algorithms on TIMER2.0. The red box highlights the cancers with consistent trends of more than two cells. **(B)** The purity and purity-adjusted correlations between JMJD8 and Tregs, CAFs, and MDSCs in 7 cancers are highlighted in panel **(A)**, presented by scatter plots from TIMER2.0. **(C)** Multiple algorithms calculated the correlations between JMJD8 and CD8+ T-cell infiltration. **(D)** The table shows the correlations between JMJD8 expression and CTL, CTL dysfunction, and risks.

Apart from M2 macrophages, we also sought other members contributing to immunosuppression. We used TIMER2.0 to display the correlations between JMJD8 and Tregs, CAFs, and MDSCs, JMJD8's positive correlations with at least two cell types were observed in several cancers, including CESC, COAD, HNSC, LUSC, PAAD, STAD, and THYM (**Figure 10A**). Their purity-adjusted correlations are presented in **Figure 10B**, showing CAFs with the highest correlations with JMJD8 in CESC, COAD, HNSC, LUSC, PAAD, and THYM.

Since CTLs were the main affected cells during immunosuppression, we also investigated JMJD8’s association with CD8+ T cells *via* 10 algorithms on TIMER2.0, and considerable negative relevance was observed in BRCA, HNSC, LUAD, and SKCM (**Figure 10C**). In addition, T-cell dysfunction was found related to JMJD8 in neuroblastoma and BRCA *via* TIDE web tool and a negative correlation was discovered between CTL and JMJD8 in BRCA (**Figure 10D**).

The extra exploration of the association between JMJD8 and other immunosuppressive cells in pan-cancer indicated that JMJD8 also functioned in CAFs, Tregs, and MDSCs and inhibited the anticancer immune by targeting CTLs.

### JMJD8 Affects Cancer Therapeutic Responses and the Molecular Docking of JMJD8-Targeted Compounds

**Figure 11:**
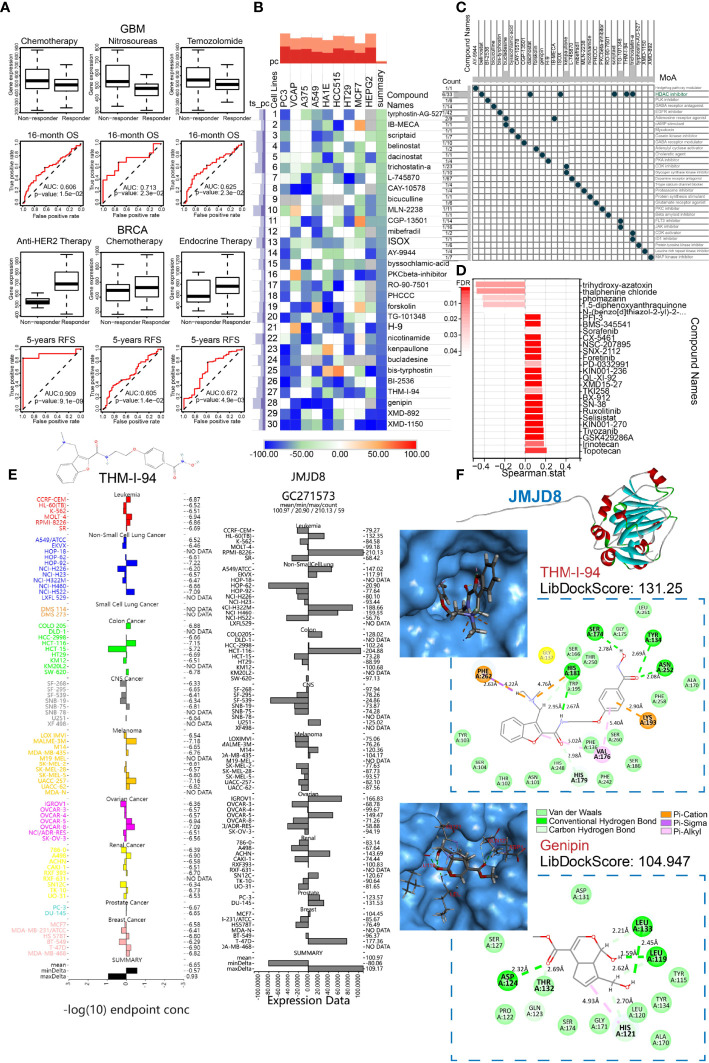
JMJD8 predicted therapeutic responses and was docked to JMJD8-targeted drugs. **(A)** Box plots show the JMJD8 expression differences between responders and non-responders, and ROC presents the predictive accuracy of patient therapeutic response by JMJD8 levels on the ROCplotter online website. **(B)** The heatmap exhibits the top 30 compounds, experimentally causing transcriptional alterations opposite to those affected by median JMJD8 expression grouping. The color bar and the block color represent the similarity scores. **(C)** The MoA scatter plots depict the MoA of the top 30 compounds in panel **(B)**; the Count column shows the ratio of the certain compound to all compounds in cMap database with the same MoA. **(D)** The bar chart shows Spearman’s correlations between mRNA alterations caused by JMJD8 grouping and by drugs from RNAactDrug, with FDR < 0.05. **(E)** The THM-I-94 GI50 (left) and JMJD8 expression of cancer cell lines were tested in the NCI60 project. The midlines represent mean −log10(GI50) or mean JMJD8 expression. **(F)** The 3D top molecular structure exhibits the built protein *via* homology modeling, and the top left images show the JMJD8 interactive pocket for drugs. The 2D graphs within the dashed line boxes present the drug’s 2D structure, interactive amino residues, molecular forces, and molecular spatial distance.

To seek whether JMJD8 can predict therapeutic responses to cancers, we obtained the data from ROCplotter to show the association between the therapeutic outcomes and JMJD8 expression in four cancer types (BRCA, OV, GBM, and CRC). In GBM, JMJD8 was highly expressed in non-responders after chemotherapy, especially post-nitrosourea treatment, and the area under the curve (AUC) value of post-nitrosourea 16-month OS reached 0.7. However, in BRCA, responders of post-anti-HER2, chemotherapy, and endocrine therapy harbored higher JMJD8 expression, with the highest AUC value of 5-year PFS prediction of anti-HER2 therapy reaching 0.909 (**Figure 11A**).

Given the poor therapeutic effects of high-JMJD8 GBM patients receiving routine chemotherapy, we attempted to identify potential anti-JMJD8 drugs with higher effects, which may also improve GBM sensitivity to current chemotherapy. The cMap tool was utilized to filter the compounds, causing opposite transcriptional alterations to those that increased by high-JMJD8 expression, in 9 different tumor cell lines and the top 30 compounds with JMJD8-targeted potential were displayed (**Figure 11B**). Notably, the MoA of six compounds was the pattern of histone deacetylase (HDAC) inhibitors, suggesting the mechanism of JMJD8 functions in cancers (**Figure 11C**). We also searched the RNAactDrug for drugs correlated to JMJD8 mRNA expression. The top 26 drugs with FDR < 0.05 were shown, while only 5 drugs were identified opposite to JMJD8 expression (**Figure 11D**).

For the top cMap compounds, we compared their GI50 after treating pan-cancer cell lines using COMPARE tools. For no testing data of XMD-1150, XMD-892, genipin was found; we exhibited the GI50s of THM-I-94 and compared them with the JMJD8 expression in pan-cancer cell lines. The average −log10(GI50) of THM-I-94 was −6.65, and in central nervous system (CNS) tumor cell lines, high JMJD8 expression corresponded to higher GI50 (**Figure 11E**). To further investigate whether these compounds can bind to JMJD8 protein, we performed homology modeling of JMJD8 protein and its molecular docking with potential drugs. With the use of alphaFold2.0, 5 models were built using the FASTA sequence listed in **Supplementary Material S5**, and the rank_1 model was retained and estimated with an Overall Quality Factor of 75.7322. Subsequently, molecular docking was conducted *via* Discovery Studio (version 4.5); XMD-1150 and XMD-892 failed to dock with JMJD8, while genipin and THM-I-94 succeeded, with the highest LibDockScore of 131.25 and 104.947, respectively. The 3D structures of the docking pockets and the 2D graph showing the interactive forces and distances are presented in **Figure 11F**. THM-I-94 interacted with JMJD8 residues by many types of interactions.

Taken together, THM-I-94 and genipin were identified as potential JMJD8-targeted drugs and may be effective for temozolomide- and nitrosourea-resistant GBMs as alternative therapies.

## Discussion

Abnormally expressed products sequentially developed cancers; these cancer-responsible products can be derived from genomic alterations, transcriptional abnormalities, post-translation, or epigenetic modulations of specific genes and affect patient prognosis *via* different mechanisms. Here, we comprehensively introduced a recently identified cancer gene, JMJD8; described its clinical significance, multi-omics characteristics, and roles in cancer immunity; and screened potential target drugs in pan-cancer. Until the recent 3 years, JMJD8 was discovered to affect tumor cell progress, but limited evidence was presented, and their trends were not consistent (9–11). Our studies showed that JMJD8 was highly expressed in GBM, LGG, and STAD and predicted shorter survival. In ESCA, PCPG, THYM, PRAD, and SARC, it indicated a better prognosis, demonstrating a tumor type-dependent factor.

DNA repair was commonly composed of MMR, HRD, and non-homologous end joining (NHEJ). DNA damage response-induced chromatin modulations triggered the DNA repair processes. Once started, the repair-related proteins were recruited in the nucleus to repair broken DNA; the HRR process can prevent cells in the S, G2, and M phases of the cell cycle (13, 52). In our study, we discovered that JMJD8 reduced TMB, MSI, and HRD possibly *via* MMR and HRR systems in some cancers like BRCA, and the two repair systems facilitated the stemness maintenance in glioma, HNSC, and OV. These results seem to suggest that JMJD8 is a crucial member of MMR and HRR, with further supportive discoveries of JMJD8, p53/RB, SWI/SNF, chromatin modifier signature correlations, and the reversely enriched G2M checkpoint pathway. Meanwhile, other JmjC domain-containing members can also modulate DNA repair processes; JMJD5 was required in HRR (53); JMDH1A suppressed NHEJ (54). The opposite effects on DNA repair by JMJD5 and JHDM1A were both mediated by H3K36 demethylation. Interestingly, Su et al. reported that JMJD8 suppressed NHEJ activity in LUAD (9), but we found that JMJD8 is associated with enhanced HRR in many cancers. Though JMJD8’s demethylation activities were not elucidated in any studies, the strong associations between JMJD8 and 4 methyltransferases were noticed in our analyses. These studies raised several questions: does the demethylation of H3K36 simultaneously promote HRR and inhibit NHEJ, or were HRR and NHEJ processes activated sequentially? If HRR and NHEJ were independently regulated, which one is predominant during the DNA double-strand break? Moreover, we found 6 JMJD8-related compounds showing HDAC inhibitors’ MoA. Since histone deacetylation is a switch of DNA repair (55), we also doubted whether JMJD8 controls the chromatin deacetylation to trigger DNA repair. To answer these questions, future efforts should be focused on finding the direct effects that JMJD8 mediates, and this study pointed the way.

Previous studies have not shown the roles of JmjC domain-containing members in cancer immune. Here, we discovered that higher JMJD8 expression is correlated to low immune infiltration, immunosuppressive cancer subtypes, and globally reduced cytokine receptors. At the same time, decreased JMJD8 expressions were observed after anticancer cytokine treatments in several cancer cells, strongly indicating an immunosuppressive role of JMJD8 in most cancers. More specifically, M2 macrophages were engaged in environment remodeling, which was validated by bulk or single-cell transcriptional sequencing data and co-expression of JMJD8 and M2 macrophage biomarkers. Given the DNA repair potential of JMJD8, these discoveries corresponded to a previous study (56) where authors noticed the roles of DNA damage repair in modulating M2 macrophage polarization, naturally leading us to the question of whether JMJD8 high expression drives or results from M2 macrophage polarization *via* DNA repair-related pathways. Nevertheless, JMJD8 seems a reliable biomarker of M2 macrophage infiltration as we have revealed. Moreover, JMJD8 was also associated with high infiltration of Tregs, CAFs, and MDSCs; low infiltration; and dysfunction of CD8+ T cells, demonstrating its broader immunosuppressive effects. To our knowledge, this is the first study showing clear associations between the JmjC domain-containing member and cancer immune.

Interestingly, we identified CD276 with an exceptionally high association with JMJD8 in 15 cancer types. CD276 is an immune checkpoint target highly expressed in cancer cells; it promotes M2 macrophage infiltration and decreases CD8+ T-cell infiltration (57). It also evaded CSCs from immune surveillance and protected them from CD8+ T-cell attacks (58). Hence, we reckoned that JMJD8 might mediate CD276-induced M2 polarization, stemness maintenance, and CD8+ T-cell inhibition. Also, JMJD8 facilitated GBM chemoresistance to DNA alkylating agents (nitrosoureas and temozolomide) probably *via* DNA repair. With these aspects in mind, we identified 2 potential JMJD8-targeted drugs with possible docking modes and expect them to be effective therapies for those who suffer chemoresistance to routine treatment.

Conclusively, we performed multi-omics pan-cancer analyses of JMJD8 and identified it as a prognostic biomarker. JMJD8 may participate in DNA repair *via* MMR or HDR to promote cancer genome stability, stemness maintenance, and chemoresistance in cancer cells. Significantly, JMJD8 was involved in cancer immunity. We confirmed it as a biomarker of M2 macrophage infiltration in various cancers and speculated that JMJD8 mediated the CSC immunity surveillance, M2 macrophage polarization, and CD8+ T-cell depression induced by CD276. Given the roles of JMJD8, we screened out potential compounds as novel therapeutic strategies. As a recently reported gene, we believed this study shed light on its functional mechanism in cancers and provided promising treatment for patients suffering from poor therapeutic effects.

## Data Availability Statement

The datasets presented in this study can be found in online repositories. The names of the repository/repositories and accession number(s) can be found in the article.

## Author Contributions

XL, QC, and CB contributed to the conception of the study. HZ, LZ, and ZW performed the experiments and interpretation of data. XL and ZD contributed significantly to the analysis and manuscript preparation. XL and XZ performed the data analyses and wrote the manuscript. JZ, PL, JH, ZL, and QC helped perform the analysis with constructive discussions. ZW, HZ, ZD, JZ, PL, LZ, JH, ZL, CB, and QC helped revise the manuscript critically. All authors contributed to the article and approved the submitted version.

## Funding

This work was supported by the National Nature Science Foundation of China [Nos.82073893 and 81703622], the Hunan Provincial Natural Science Foundation of China [No.2022JJ20095], the Hunan Provincial Health Committee Foundation of China [No.202204044869], and Xiangya Hospital Central South University postdoctoral foundation.

## Conflict of Interest

The authors declare that the research was conducted in the absence of any commercial or financial relationships that could be construed as a potential conflict of interest.

## Publisher’s Note

All claims expressed in this article are solely those of the authors and do not necessarily represent those of their affiliated organizations, or those of the publisher, the editors and the reviewers. Any product that may be evaluated in this article, or claim that may be made by its manufacturer, is not guaranteed or endorsed by the publisher.

## Supplementary Material

Supplementary Material S1 The hazard ratios of JMJD8 in pan-cancer. **(A–C)** The forest plots of JMJD8’s hazard ratios in predicting overall survival **(A)**, disease-specific survival **(B)**, and progression-free interval **(C)**.

Supplementary Material S2 Clinical-related alternative splicing events of JMJD8. **(A)** The tables of reads-in, reads-out, PSI differences in tumor, adjacent, normal tissues of JMJD8 alt_3prime_91164, alt_3prime_91166, ES_32952, ES_119015 alternative splicing events. **(B)** The PSI differences between tumor-normal, tumor-adjacent tissues, and log2(Hazard Ratio) of OS, PFI in pan-cancer. PSI, Percent Spliced In; alt_3, 3’ terminal alternate; ES, exon skip; GTEx, Genotype-Tissue Expression; OS, overall survival; DSS, disease-specific survival; PFI, progression-free interval.

Supplementary Material S3 Expression correlations between JMJD8 and DNA repair-related pathway signature **(A–C)** The expression correlations between JMJD8 and SWI/SNF **(A)**, p53/Rb **(B)**, Chromatin modifiers **(C)** pathway signatures in BRCA, HNSC, GBM. BRCA, breast cancer; HNSC, head and neck squamous cell carcinoma; GBM, glioblastoma.

Supplementary Material S4 HE staining photos of the pan-cancer sections. The photos show the HE staining images of pan-cancer **(A–H)** sections corresponding to the immunofluorescence images in **Figure 9**.

## Abbreviations

LAML, acute myeloid leukemia; ACC, adrenocortical carcinoma; BLCA, bladder urothelial carcinoma; LGG, brain lower-grade glioma; BRCA, breast invasive carcinoma; CESC, cervical squamous cell carcinoma and endocervical adenocarcinoma; CHOL, cholangiocarcinoma; LCML, chronic myelogenous leukemia; COAD, colon adenocarcinoma; CNTL, controls; ESCA, esophageal carcinoma; GBM, glioblastoma multiforme; HNSC, head and neck squamous cell carcinoma; KICH, kidney chromophobe; KIRC, kidney renal clear cell carcinoma; KIRP, kidney renal papillary cell carcinoma; LIHC, liver hepatocellular carcinoma; LUAD, lung adenocarcinoma; LUSC, lung squamous cell carcinoma; DLBC, lymphoid neoplasm diffuse large B-cell lymphoma; MESO, mesothelioma; MISC, miscellaneous; OV, ovarian serous cystadenocarcinoma; PAAD, pancreatic adenocarcinoma; PCPG, pheochromocytoma and paraganglioma; PRCA, prostate carcinoma; PRAD, prostate adenocarcinoma; READ, rectum adenocarcinoma; SARC, sarcoma; SKCM, skin cutaneous melanoma; STAD, stomach adenocarcinoma; TGCT, testicular germ cell tumors; THYM, thymoma; THCA, thyroid carcinoma; UCS, uterine carcinosarcoma; UCEC, uterine corpus endometrial carcinoma; UVM, uveal melanoma; TARGET-WT, TARGET-Wilms tumor of kidney tumors; UCSC, University of California, Santa Cruz; AEL, acute erythroblastic leukemia; PUC, papillary ureter carcinoma; RPUC, renal pelvis urothelial cell carcinoma; UCC, ureter urothelial cell carcinoma; TCGA, The Cancer Genome Atlas; TARGET, Therapeutically Applicable Research to Generate Effective Treatments; GEO, Gene Expression Omnibus; GTEx, Genotype-Tissue Expression; JmjC, Jumonji C; HRR, homologous recombination repair; MMR, mismatch repair; TME, tumor microenvironment; SNV, simple-nucleotide variation; NCBI, National Center for Biotechnology Information; UPTAC, Clinical Proteomic Tumor Analysis Consortium; TIDE, Tumor Immune Dysfunction and Exclusion; CNV, copy number variations; TMB, tumor mutation burden; MIS, microsatellite instability; HRD, homologous recombination deficiency; DMPsi, differentially methylated probes-based stemness index; CTL, cytotoxic T lymphocyte; m1A, N1‐methyladenosine; m5C, 5‐methylcytosine; m6A, N6‐methyladenosine; AS, alternative splicing; PSI, percent spliced-in; GO, Gene Ontology; GSEA, Gene Set Enrichment Analysis; KEGG, Kyoto Encyclopedia of Genes and Genomes; ESTIMATE, Estimation of STromal and Immune cells in MAlignant Tumour tissues using Expression data; TISMO, Tumor Immune Syngeneic Mouse; TISCH, Tumor Immune Single-cell Hub; TSA, tyramide signal amplification; DAPI, 4′,6-Diamidino2-phenylindole dihydrochloride; Tregs, regulatory T cells; CAFs, cancer-associated fibroblasts; MDSCs, myeloid-derived suppressor cells; ROC, operating characteristic curves; MoA, mechanisms of action; GI50, 50% growth inhibition; DTP, Developmental Therapeutics Program; NCI, National Cancer Institute; OS, overall survival; DSS, disease-specific survival; PFI, progression-free interval; DFI, disease-free interval; AUC, area under the curve; NHEJ, non-homologous end joining

## Glossary

**Table:**

LAML	acute myeloid leukemia
ACC	adrenocortical carcinoma
BLCA	bladder urothelial carcinoma
LGG	brain lower-grade glioma
BRCA	breast invasive carcinoma
CESC	cervical squamous cell carcinoma and endocervical adenocarcinoma
CHOL	cholangiocarcinoma
COAD	colon adenocarcinoma
ESCA	esophageal carcinoma
GBM	glioblastoma multiforme
HNSC	head and neck squamous cell carcinoma
KICH	kidney chromophobe
KIRC	kidney renal clear cell carcinoma
KIRP	kidney renal papillary cell carcinoma
LIHC	liver hepatocellular carcinoma
LUAD	lung adenocarcinoma
LUSC	lung squamous cell carcinoma
DLBC	lymphoid neoplasm diffuse large B-cell lymphoma
MESO	mesothelioma
OV	ovarian serous cystadenocarcinoma
PAAD	pancreatic adenocarcinoma
PCPG	pheochromocytoma and paraganglioma
PRCA	prostate carcinoma
PRAD	prostate adenocarcinoma
READ	rectum adenocarcinoma
SARC	sarcoma
SKCM	skin cutaneous melanoma
STAD	stomach adenocarcinoma
TGCT	testicular germ cell tumors
THYM	thymoma
THCA	thyroid carcinoma
UCS	uterine carcinosarcoma
UCEC	uterine corpus endometrial carcinoma
UVM	uveal melanoma
TARGET-WT	TARGET-Wilms tumor of kidney tumors
UCSC	University of California Santa Cruz
AEL	acute erythroblastic leukemia
PUC	papillary ureter carcinoma
RPUC	renal pelvis urothelial cell carcinoma
UCC	ureter urothelial cell carcinoma
TCGA	The Cancer Genome Atlas
TARGET	Therapeutically Applicable Research to Generate Effective Treatments
GEO	Gene Expression Omnibus
GTEx	Genotype-Tissue Expression
JmjC	Jumonji C
HRR	homologous recombination repair
MMR	mismatch repair
TME	tumor microenvironment
SNV	simple-nucleotide variation
NCBI	National Center for Biotechnology Information
UPTAC	Clinical Proteomic Tumor Analysis Consortium
TIDE	Tumor Immune Dysfunction and Exclusion
CNV	copy number variations
TMB	tumor mutation burden
MIS	microsatellite instability
HRD	homologous recombination deficiency
DMPsi	differentially methylated probes-based stemness index
CTL	cytotoxic T lymphocyte
m1A	N1–methyladenosine
m5C	5–methylcytosine
m6A	N6–methyladenosine
AS	alternative splicing
PSI	percent spliced-in
GO	Gene Ontology
GSEA	Gene Set Enrichment Analysis
KEGG	Kyoto Encyclopedia of Genes and Genomes
ESTIMATE	Estimation of STromal and Immune cells in MAlignant Tumour tissues using Expression data
TISMO	Tumor Immune Syngeneic Mouse
TISCH	Tumor Immune Single-cell Hub
TSA	tyramide signal amplification
DAPI	4′ 6-Diamidino2-phenylindole dihydrochloride
Tregs	regulatory T cells
CAFs	cancer-associated fibroblasts
MDSCs	myeloid-derived suppressor cells
ROC	operating characteristic curves
MoA	mechanisms of action
GI50	50% growth inhibition
DTP	Developmental Therapeutics Program
NCI	National Cancer Institute
OS	overall survival
DSS	disease-specific survival
PFI	progression-free interval
DFI	disease-free interval
AUC	area under the curve
NHEJ	non-homologous end&nbsp;joining
