# Supplementary figures and images for: JMJD8 Is an M2 Macrophage Biomarker, and It Associates With DNA Damage Repair to Facilitate Stemness Maintenance, Chemoresistance, and Immunosuppression in Pan-Cancer
Source: Front Immunol. 2022 Jul 11;13:875786. doi: 10.3389/fimmu.2022.875786 (PMC9309472; doi:10.3389/fimmu.2022.875786)

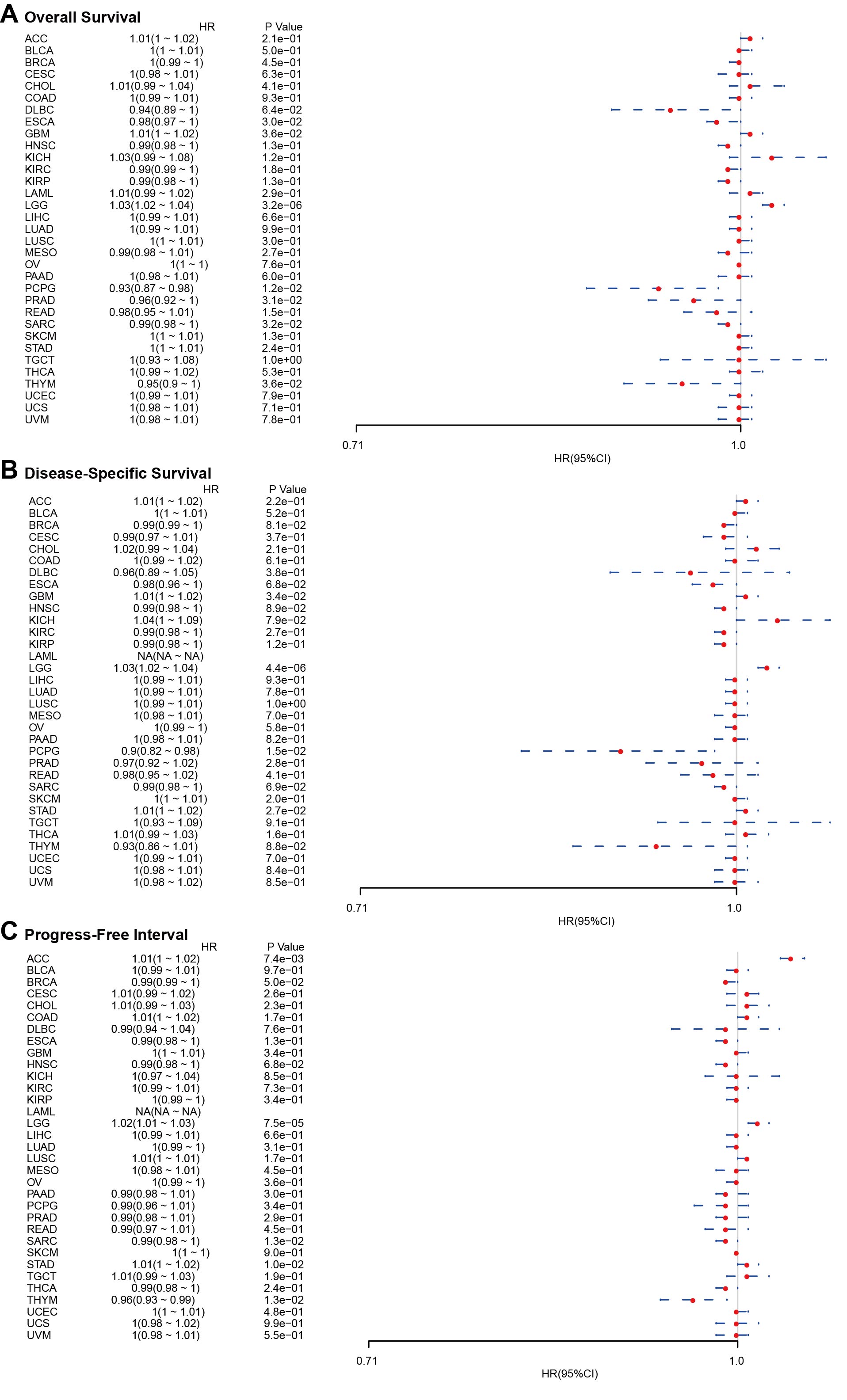

Supplement: Supplementary Material S1 — The hazard ratios of JMJD8 in pan-cancer. (A–C) The forest plots of JMJD8’s hazard ratios in predicting overall survival (A), disease-specific survival (B), and progression-free interval (C). [file Image_1.jpeg]

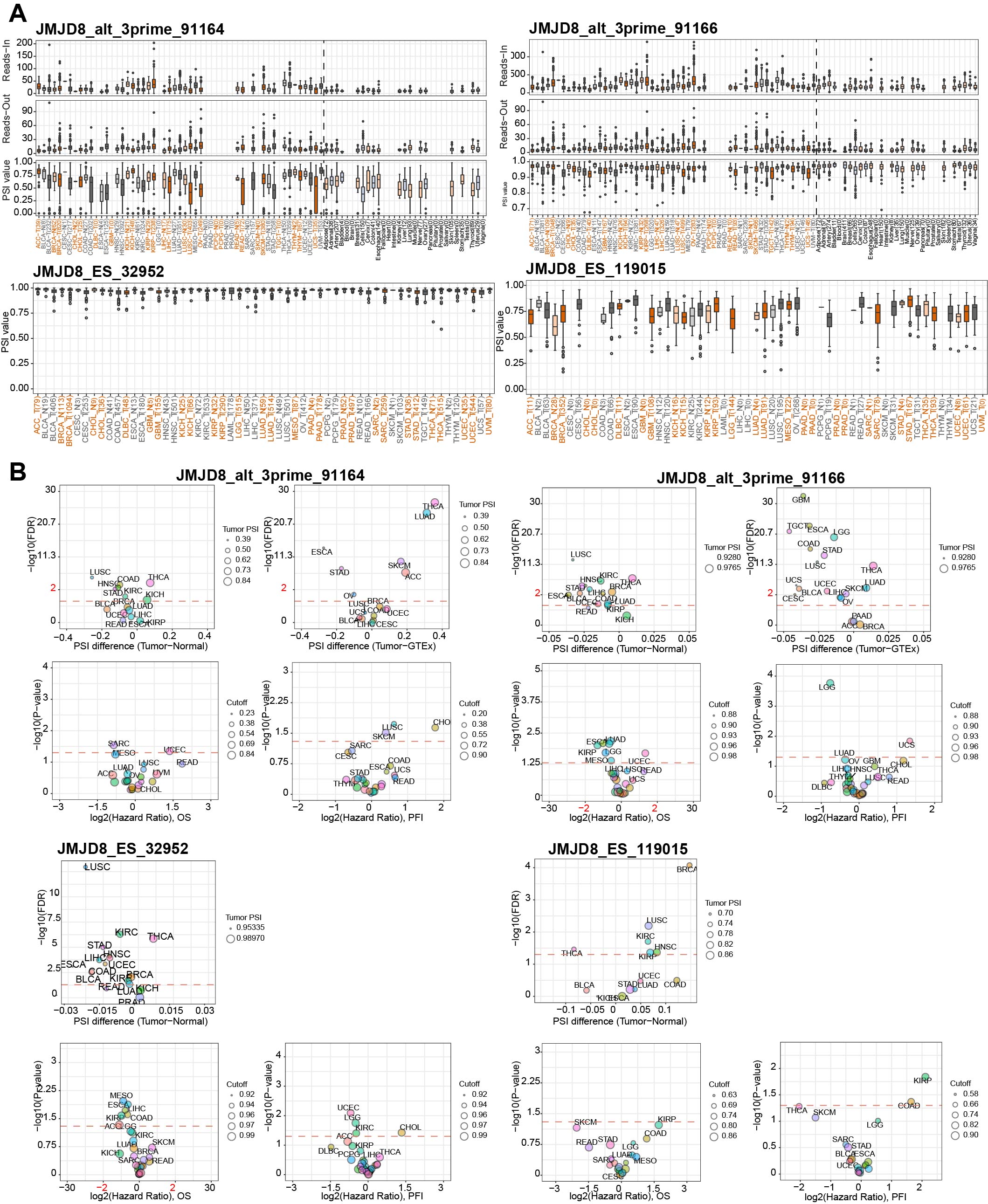

Supplement: Supplementary Material S2 — Clinical-related alternative splicing events of JMJD8. (A) The tables of reads-in, reads-out, PSI differences in tumor, adjacent, normal tissues of JMJD8 alt_3prime_91164, alt_3prime_91166, ES_32952, ES_119015 alternative splicing events. (B) The PSI differences between tumor-normal, tumor-adjacent tissues, and log2(Hazard Ratio) of OS, PFI in pan-cancer. PSI, Percent Spliced In; alt_3, 3’ terminal alternate; ES, exon skip; GTEx, Genotype-Tissue Expression; OS, overall survival; DSS, disease-specific survival; PFI, progression-free interval. [file Image_2.jpeg]

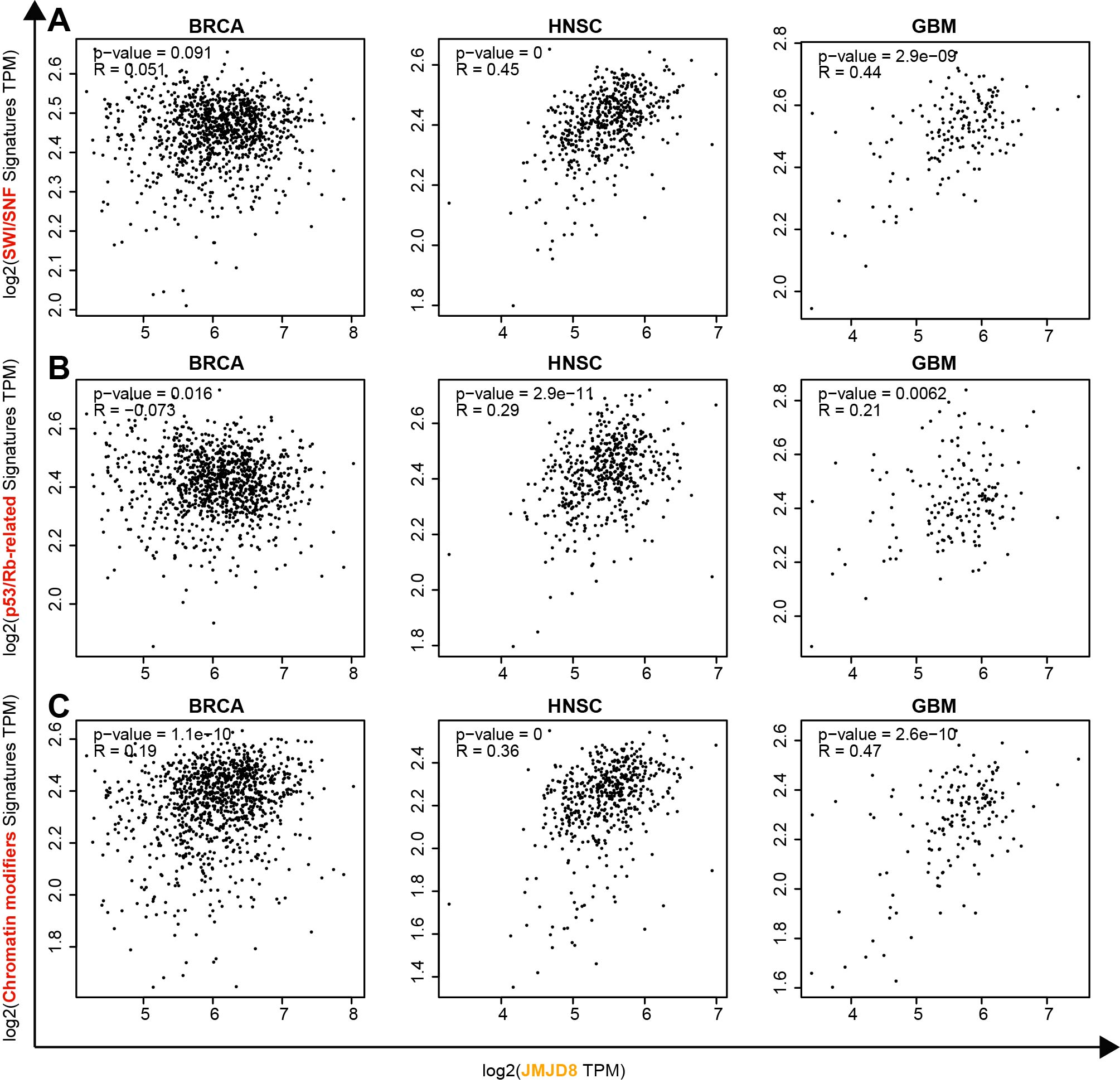

Supplement: Supplementary Material S3 — Expression correlations between JMJD8 and DNA repair-related pathway signature (A–C) The expression correlations between JMJD8 and SWI/SNF (A), p53/Rb (B), Chromatin modifiers (C) pathway signatures in BRCA, HNSC, GBM. BRCA, breast cancer; HNSC, head and neck squamous cell carcinoma; GBM, glioblastoma. [file Image_3.jpeg]

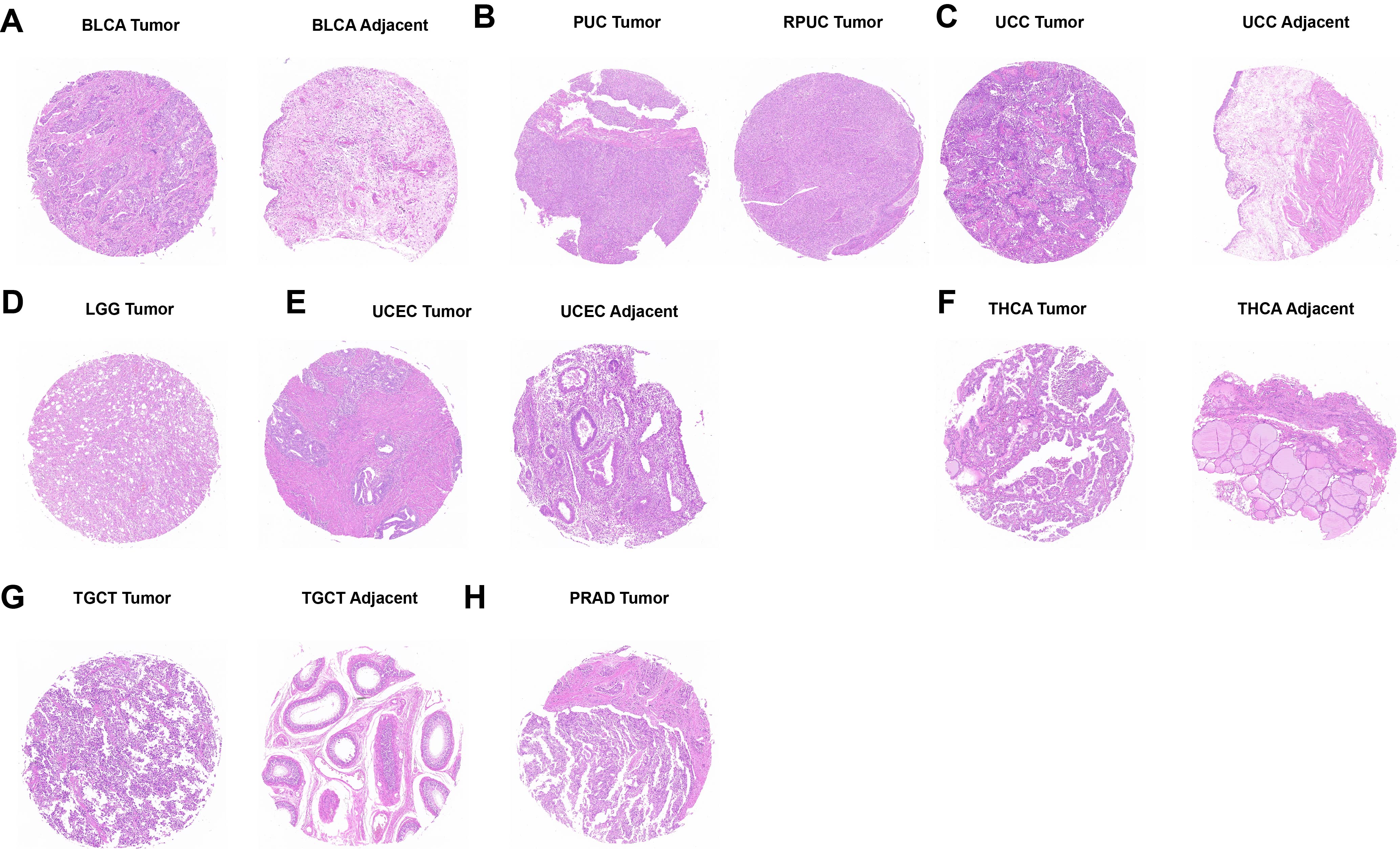

Supplement: Supplementary Material S4 — HE staining photos of the pan-cancer sections. The photos show the HE staining images of pan-cancer (A–H) sections corresponding to the immunofluorescence images in Figure 9. [file Image_4.jpeg]
