# Supplementary material for: JMJD8 Is an M2 Macrophage Biomarker, and It Associates With DNA Damage Repair to Facilitate Stemness Maintenance, Chemoresistance, and Immunosuppression in Pan-Cancer
Source: Front Immunol. 2022 Jul 11;13:875786. doi: 10.3389/fimmu.2022.875786 (PMC9309472; doi:10.3389/fimmu.2022.875786)
Supplement: Supplementary file 5 [file DataSheet_1.docx]

**Supplementary Material S5 The FASTA sequence used for JMJD8 protein homology modeling.**

>NP_001005920.3 jmjC domain-containing protein 8 isoform 1 precursor [Homo sapiens]

MAPASRLLALWALAAVALPGSGAEGDGGWRPGGPGAVAEEERCTVERRADLTYAEFVQQYAFVRPVILQGLTDNSRFRALCSRDRLLASFGDRVVRLSTANTYSYHKVDLPFQEYVEQLLHPQDPTSLGNDTLYFFGDNNFTEWASLFRHYSPPPFGLLGTAPAYSFGIAGAGSGVPFHWHGPGYSEVIYGRKRWFLYPPEKTPEFHPNKTTLAWLRDTYPALPPSARPLECTIRAGEVLYFPDRWWHATLNLDTSVFISTFLG
